# In silico discovery of nanobody binders to a G-protein coupled receptor using AlphaFold-Multimer

**DOI:** 10.1038/s41467-026-72093-5

**Published:** 2026-04-23

**Authors:** Edward P. Harvey, Jeffrey S. Smith, Joseph D. Hurley, Alyana J. Granados, Ernst W. Schmid, Jason G. Liang-Lin, Huyang Zhang, Emily M. Meara, Elizabeth K. Wren, Steffanie Paul, Matthew P. Ferguson, Victor G. Calvillo-Miranda, Miguel A. Alcantar, Debora S. Marks, Johannes C. Walter, Andrew C. Kruse, Katherine J. Susa

**Affiliations:** 1 Department of Biological Chemistry and Molecular Pharmacology, Blavatnik Institute, Harvard Medical School, Boston, MA USA; 2 Department of Dermatology, Brigham and Women’s Hospital, Boston, MA USA; 3 Department of Pharmaceutical Chemistry, University of California, San Francisco, CA USA; 4 Department of Systems Biology, Harvard Medical School, Boston, MA USA; 5 Broad Institute of Harvard and MIT, Cambridge, MA USA; 6 Department of Biomedical Engineering, University of California, Irvine, CA USA; 7 Howard Hughes Medical Institute, Boston, MA USA

**Keywords:** Bioinformatics, Applied immunology, G protein-coupled receptors, Computational models, Computational biophysics

## Abstract

Antibodies are central mediators of the adaptive immune response, and they are powerful research tools and therapeutics. Antibody discovery requires substantial experimental effort, such as immunization campaigns or in vitro library screening. Predicting antibody-antigen binding a priori remains challenging. However, recent machine learning methods raise the possibility of in silico antibody discovery, bypassing or reducing initial experimental bottlenecks. Here, we report a virtual screen using AlphaFold-Multimer (AF-M) that prospectively identified nanobody binders to MRGPRX2, a G protein-coupled receptor (GPCR) and therapeutic target for the treatment of pseudoallergic inflammation and itch. Using previously reported nanobody-GPCR structures, we identified a set of AF-M outputs that effectively discriminate between interacting and non-interacting nanobody-GPCR pairs. We used these outputs to perform a prospective in silico screen, identified nanobodies that bind MRGPRX2 with high affinity, and confirmed activity in signaling and functional cellular assays. Our results provide a proof of concept for fully computational antibody discovery pipelines that can circumvent laboratory experiments.

## Abstract (web summary)

Antibody discovery often requires major experimental effort. Here, authors report a virtual screen using AlphaFold-Multimer that identified nanobody binders to MRGPRX2, a therapeutic target for itch, demonstrating fully in silico antibody discovery.

## Introduction

Antibodies are proteins produced by B cells that recognize foreign antigens to protect against infection and are increasingly valuable as research tools and therapeutics^1^. Antibodies now make up the fastest growing class of drugs, with more than 100 antibodies approved as drugs and more than 800 in clinical trials as of 2020^2^. Antibodies can selectively differentiate between proteins that are highly similar in structure, and they can be engineered to have slow off-rates and favorable pharmacokinetic properties, permitting infrequent drug dosing^3^. Nanobodies are single-domain, heavy-chain-only camelid antibody fragments and are particularly versatile due to their size, simplicity, and biochemical tractability. Nanobodies are an emerging class of antibody drugs, with one FDA-approved nanobody^4^ and others in clinical development^5^.

Most FDA-approved monoclonal antibody therapeutics were discovered using immunization technology developed in the 1970s^6^. However, many putative drug targets possess a high degree of sequence conservation in mammals, leading to immunization failures. To overcome this challenge, yeast surface display and phage display libraries provide methods to discover new synthetic antibody binders. However, these methods require specialized equipment and techniques and often generate polyreactive binders due to the lack of in vivo immune filtering^7^.

The recent development of AlphaFold2 and its successors has revolutionized computational and structural biology by enabling accurate predictions of protein structures and multi-protein complexes from sequence inputs^8–12^. AlphaFold2 is trained on both structural data in the Protein Databank (PDB) and Multiple Sequence Alignments (MSAs) that contain co-evolutionary information about physically interacting amino acid residues. Despite the lack of co-evolution between antibodies and antigens, we sought to investigate the possibility that AlphaFold-Multimer (AF-M), a related method trained specifically on protein complexes, can predict antibody binding to protein targets based solely on structural data in the PDB. We focused on nanobodies, which we reasoned would be ideal candidates for computational screening due to their single-chain structure that lacks a light chain region and the large number of nanobody-target complexes deposited in the PDB.

Computational antibody discovery methods raise the exciting possibility of identifying selective antibody binders completely in silico, circumventing challenges associated with immunization and library display selections. Computational methods like RFdiffusion have recently been reported to identify single-domain antibody fragment binders to protein targets^13,14^. However, this method performs best when structural information about similar antigen/antibody binding complexes is available and requires user-specified epitopes^13,14^.

Here, we find that AF-M reliably discriminates known GPCR-binding nanobodies from non-binding controls, even with careful efforts to separate training and test sets. Moreover, in a prospective screen of an in silico nanobody library of 10,000 nanobodies, we identified binders ranging in affinity from 20 to 200 nanomolar to MRGPRX2, a GPCR involved in non-canonical mast cell degranulation and a potential drug target for the treatment of certain inflammatory conditions. Our findings suggest that similar in silico antibody discovery approaches may be useful to circumvent time-consuming experimental selection campaigns and more expeditiously identify antibody binders to cellular surface receptors, particularly GPCRs.

## Results

### AlphaFold-Multimer accurately predicts nanobody binding to GPCRs

**Fig. 1:**
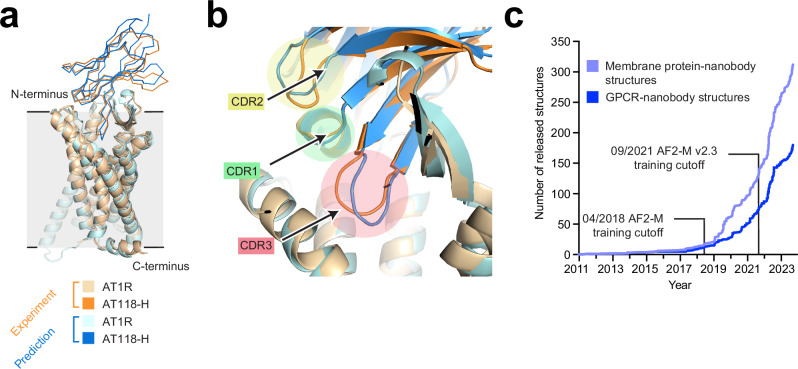
AlphaFold-Multimer accurately predicts an unusual binding pose and structure of a nanobody with a GPCR. **a** Overlay of the experimentally determined structure of the complex of the GPCR AT1R with nanobody AT118-H (PDB 8TH3) and the AF-M prediction, demonstrating nearly identical structural models. **b** The CDR regions of AT118-H in the experimentally acquired and computationally predicted structural models are closely aligned. **c** The number of released nanobody structures bound to either membrane proteins or GPCRs has increased rapidly. Source data are provided as a Source Data file.

AlphaFold2 and its successor AlphaFold-Multimer were trained using both sequence and structural data to generate structural predictions. By analyzing co-evolutionary patterns across homologous protein sequences in a multiple sequence alignment (MSA), residue interactions that contribute to structural stability or function can be inferred. This co-evolutionary signal, in combination with experimentally determined protein structures, has allowed AlphaFold2 and related methods to achieve near-experimental accuracy for many proteins^10–12^. However, the performance of these models is notably diminished for antibody-antigen pairs, as these pairs do not meaningfully co-evolve, and the models must rely exclusively on patterns in the available protein structure data. Despite this limitation, we observed that AlphaFold-Multimer could accurately predict the unusual structure of the GPCR angiotensin II type 1 receptor (AT1R) bound to a synthetic nanobody, AT118-H (Fig. 1a, b). Importantly, this experimentally determined cryo-EM structure was deposited to the PDB after the model’s training cutoff date^15^. The strong agreement between the predicted and experimentally determined structures (RMSDs: overall = 2.9 Å; receptors = 2.9 Å; nanobodies = 1.8 Å; CDR1 = 1.52 Å, CDR2 = 2.65 Å, CDR3 = 1.54 Å) is especially surprising because AT118-H induces an unusual, previously unseen conformation of AT1R in which the external side of the receptor is in an active-like state while the internal side of the receptor is in an inactive-like state^15^. The correct prediction of this unusual state shows that AF-M infers nuanced structural features rather than merely recapitulating nanobody-GPCR structures in its training data. Furthermore, AF-M’s confidence metrics for this interaction pair are quite high, as reflected by the low AT1R/AT118-H interface predicted aligned error (PAE) value (Supplementary Fig. 1C). In recent years, the number of structures of nanobodies bound to GPCRs has increased rapidly, growing from a handful in 2015 to more than 150 deposited structures in 2023 (Fig. 1c, and Supplementary Fig. 1A, B). This might, in part, explain why AF-M correctly predicted the structure of AT1R bound to AT118-H, while also raising the possibility of continued improvements in the future.

The surprising ability of AF-M to accurately predict the structure of the AT1R/AT118-H complex led us to ask how generalizable this result might be. Specifically, we sought to test whether AF-M could distinguish true nanobody binders of GPCRs from non-binding controls. We also sought to assess performance on non-GPCR membrane proteins and soluble protein targets. We compiled sets of known nanobody-antigen pairs that were supported either by direct binding data or experimentally solved structures deposited to the PDB after the AF-M v2.3 training cutoff date of 9-30-2021 (Supplementary Data 1–3). Our training sets include 32 validated GPCR nanobody binders and 127 GPCR non-binder pairs, 17 validated membrane protein nanobody binders and 376 membrane protein non-binder pairs, and 49 validated soluble protein nanobody binders and 1469 soluble protein non-binder pairs (Supplementary Data 1–3). To generate sets of non-binding pairs, we permuted nanobody-antibody pairs such that nanobodies were paired with antigens other than their reported antigen. We confirmed that antigens included in the benchmarking sets were not too closely related to one another in sequence space through BLAST sequence homology searches. To perform predictions in a high-throughput manner, we utilized a modified AF-M ColabFold script optimized for faster prediction speed and decreased memory storage requirements^8,16,17^, and we generated structural predictions of these curated nanobody-antigen pairs to investigate which outputs, if any, were predictive of nanobody binding.

We then developed a parser that extracted or calculated metrics that we hypothesized might differ between interacting and non-interacting pairs (Table S2). Several global metrics were calculated (e.g., average predicted template modeling score (pTM) across the five AF-M models). Additionally, we filtered for residue pairs near the nanobody-target interface (defined as intra-chain residue pairs for which the Cα atoms were ≤10 Å from each other) and used these data to calculate several interface-specific metrics. Among these were average interface predicted aligned error (PAE), average interface predicted local distance difference test (pLDDT), pDockQ^18^,and average model support (the average number of models in which interface contacts are observed)^16,18^. In addition to metrics averaged across all five models, metrics derived solely from the highest confidence model (as ranked by AF-M) were also considered.

**Fig. 2:**
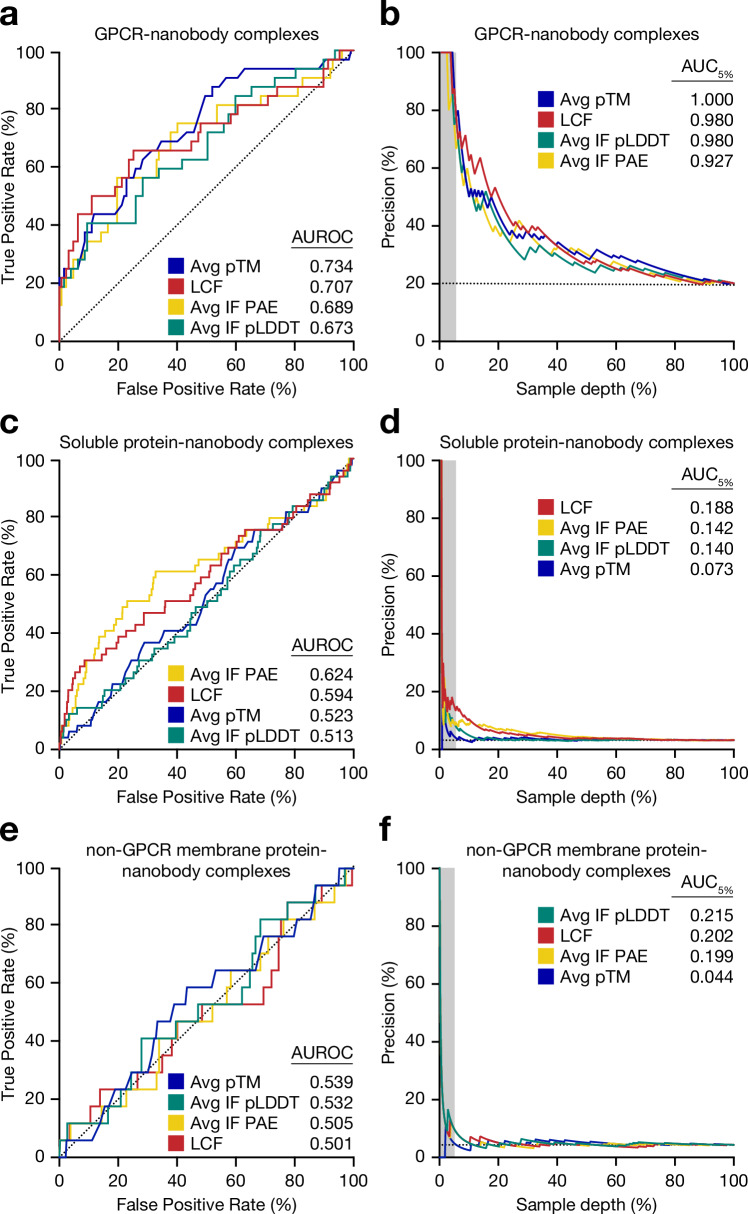
AF-M metrics differentiate between validated nanobody GPCR binders and negative controls. **a** Receiver operating characteristic curves comparing the ability of select AF-M confidence metrics and the Linear Combination Feature (LCF) to differentiate between true nanobody GPCR binders and negative controls. **b**. Comparison of AUC_5%_ values for AF-M confidence metrics and the LCF, illustrating performance specifically on highest ranked nanobodies. AF-M confidence metrics and the LCF possess poor ability to differentiate between true nanobody soluble protein binders and negative controls (**c**, **d**) and true nanobody non-GPCR membrane protein binders and negative controls (**e**, **f**). Source data are provided as a Source Data file.

We found that several of these metrics were predictive of true binding in our GPCR-nanobody dataset (Fig. 2a, Supplementary Table 2, and Supplementary Fig. 8). In particular, seven metrics had areas under the receiver operating characteristic curve (AUROCs) above 0.65: average pTM across five models (AUROC = 0.73), best model pTM (AUROC = 0.71), average interface PAE (AUROC = 0.69), best model interface PAE (AUROC = 0.68), average interface pLDDT (AUROC = 0.67), best model interface pLDDT (AUROC = 0.65), and best model pDockQ (AUROC = 0.66). We combined these features (except best model pDockQ, which is largely derived from best model pLDDT and therefore redundant) by scaling them to span the range 0–1 and calculating the product of all six scaled features to generate a Linear Combination Feature (LCF) (Supplementary Table 3). This combination feature had an AUROC of 0.71, which is slightly lower than that of its highest component feature. However, since all six component features showed nearly equivalent performance, we reasoned combining them may be more robust to new data than a single feature chosen from a modestly sized benchmarking set.

In addition to GPCR-nanobody complexes, we also assessed the performance of AF-M in distinguishing true nanobody binders from negative controls in the contexts of non-GPCR membrane protein-nanobody pairs and soluble protein-nanobody pairs. In contrast to GPCR-nanobody pairs, AF-M has low performance for these target classes, evidenced by the previously described metrics having AUROCs close to 0.5 (Fig. 2c, e). We hypothesize that this difference may be a consequence of the large number of GPCR-nanobody structures in the PDB and the relatively stereotyped binding poses that nanobodies use to engage GPCRs.

Recently, AlphaFold3 was reported to possess an improved ability to predict antibody binding to proteins^9^. We used our benchmarking sets to assess the predictive ability of AlphaFold3. Using a single initialization seed, the model achieved an AUROC of 0.74 for best model ipTM metric for the GPCR benchmarking set (Supplementary Fig. 2A) and an AUROC of 0.66 for best model ipTMs for the soluble protein benchmarking set (Supplementary Fig. 2B). For the non-GPCR membrane protein set, however, we found that AlphaFold3 best model ipTM metrics are not currently able to differentiate between true nanobody binders and negative controls (Supplementary Fig. 2C). Finally, we assessed ESMfold, an orthogonal protein structural prediction model, and found that ESMFold is unable to differentiate between true positive binding interactions and negative control binding pairs for our GPCR benchmarking dataset (Supplementary Fig. 2D–F)^19^. These results show that AF-M is currently more proficient at predicting nanobody binding to GPCRs than ESMFold.

The ability of AF-M to accurately rank binding vs. non-binding GPCR nanobodies raised the possibility that AF-M could be used as a virtual screening tool for antibody discovery. In such a screen, presumably only highly-ranked hits would be selected for further experimental validation. Therefore, model precision (i.e., the proportion of the model’s “positive” classifications that are truly positive) for the highest-ranked nanobodies is more pertinent than model accuracy for the average nanobody in the dataset, which is reflected by metrics like AUROC. To assess performance specifically on the highest ranked nanobodies, we calculated precision as a function of sample depth (the assessed proportion of the distribution of predictions ranked from highest to lowest according to their AF-M confidence metrics) and calculated AUC_5%_ values, defined as the precision for only the highest-ranked 5% of nanobodies by a given metric (Fig. 2b, d, f). The linear combination feature and each of its component metrics exhibited stellar AUC_5%_ values for the GPCR benchmarking dataset (ranging from 0.93 to 1). However, the AUC_5%_ metrics for the soluble and membrane protein benchmarking sets were much lower (≤0.22). These results suggest that AF-M could currently be deployed for in silico discovery of nanobody binders to GPCRs, but not soluble or non-GPCR membrane proteins.

### In silico discovery of MRGPRX2 nanobodies

**Fig. 3:**
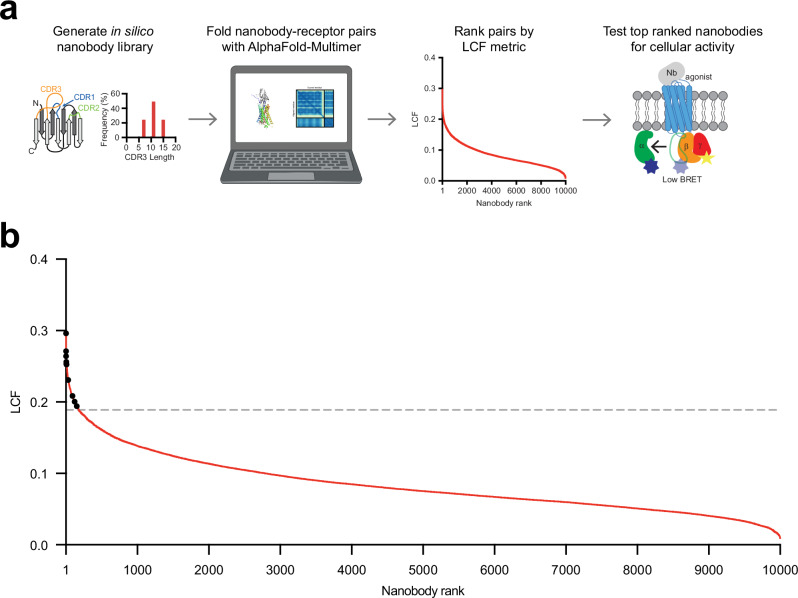
Deploying AF-M in silico screening for nanobody binders to the GPCR MRGPRX2. **a** Flowchart describing the in silico nanobody screening process. Ten thousand nanobody sequences that met prior library design specifications (McMahon et al. 2018) were computationally co-folded with the GPCR MRGPRX2 in AF-M. Nanobodies were then ranked by the LCF metric. A sample of the highest ranked nanobodies were expressed, purified, and tested for binding and activity in signaling assays. Created in BioRender. Smith, J. (2026) https://BioRender.com/jelohrd. **b** Co-folding ten thousand nanobodies with the GPCR MRGPRX2 in AF-M generated 179 nanobody-receptor pairs with LCF values greater than that of the highest value in the non-binding GPCR-nanobody set (indicated by dashed gray line). Black circles indicate nanobodies prioritized for validation studies.

To perform a virtual nanobody screen to find MRGPRX2 binders, we generated ten thousand simulated nanobody sequences that match the design parameters of our previously published nanobody yeast display library^20^ (Fig. 3a, and Supplementary Data 4). This virtual nanobody library featured three different CDR3 lengths (7, 11, and 15 residues long), and amino acids at each CDR position were sampled independently from amino acid distributions matching our previously published library. At the majority of CDR positions, all amino acids except for cysteine and methionine were sampled, and at the remainder of positions, more limited sets of amino acids were sampled, consistent with our previously published library design^20^. Importantly, this library was designed to be naive and was not intentionally enriched or filtered for GPCR-binding sequences; it also did not contain any sequences from our validation datasets and did not intentionally contain any previously known GPCR binders. As our candidate target GPCR, we selected MAS-related GPR family member X2 (MRGPRX2), a GPCR that regulates IgE-independent mast cell degranulation. This receptor was chosen because its shallow binding pocket suggests a more forgiving thermodynamic landscape relative to other GPCRs^21^. MRGPRX2 is found exclusively in primates and has low sequence similarity to MRGPRX1, MRGPRX3, and MRGPRX4, which may reduce certain contributions from other GPCRs within the PDB to the AF-M training algorithm. Currently, no FDA-approved medications target MRPGRX2, making it an attractive therapeutic target.

We predicted 10,000 MRGPRX2-nanobody structures using AF-M without templates to prevent AF-M from being unduly biased by a single structure and subsequently ranked the predictions according to our LCF metric. Importantly, structures of MRGPRX2 were deposited to the PDB several months after the AF-M training cutoff, so specific structural details of MRGPRX2 were not available a priori^22,23^. Nanobodies that possessed potential liabilities, such as glycosylation sites within the CDRs or CDR sequences predicted to be polyreactive were removed^7^. Out of the 10,000 nanobodies that we generated structural models for, 25% had potential liabilities. 179 nanobodies (1.79% of total simulated nanobodies) demonstrated compelling LCF metrics, i.e., an LCF greater than the highest LCF of the negative control nanobodies in the GPCR nanobody benchmarking dataset (Fig. 3b). We aimed to identify extracellular binders that allowed us to modulate MRGPRX2 signaling and compete with endogenous ligands, and 177 out of 179 of our “above-threshold” hits were predicted to bind extracellularly. The top six ranked nanobodies with no liabilities were expressed and purified as Fc fusions (Supplementary Table 1, and Supplementary Fig. 3). In addition, four lower-ranked nanobodies distributed evenly throughout the top 179 were chosen for expression and purification to broadly sample the population of in silico hits. Importantly, only one top-ranked nanobody was excluded from recombinant expression and purification due to a potential liability.

**Fig. 4:**
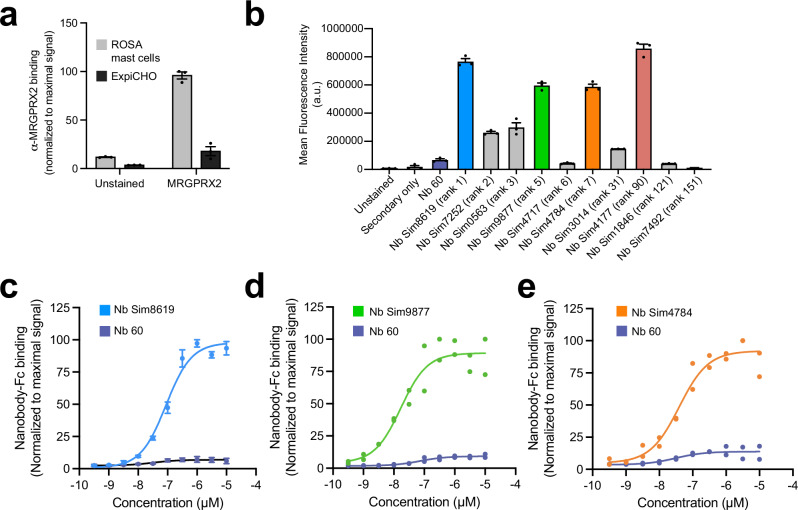
Binding of candidate MRGPRX2 nanobodies to mast cells. **a** MRGPRX2 expression levels were assessed in ROSA mast cells expressing MRGPRX2 and ExpiCHO cells lacking MRGPRX2 using a commercial anti-MRGPRX2 antibody (Biolegend Cat# 359005, AB_2750139). **b** Flow cytometry screen of binding of top-ranked clones to ROSA mast cells. Predicted binding poses of the ten experimentally tested nanobodies are shown in Supplementary Fig. 6B–D, K–Q and Supplementary Data 5. **c** Nanobody Sim8619 (rank 1), **d** nanobody Sim9877 (rank 5), **e** nanobody Sim4784 (rank 7) binding to ROSA mast cells relative to Nb 60 negative control. **a**–**c** error bars represent mean ± SEM of three biological replicates. **d**, **e** experiments were performed in biological duplicate. A representative gating strategy is shown in Supplementary Fig. 7. Source data are provided as a Source Data file.

We first screened 10 purified candidate nanobodies for binding to an immortalized mast cell line (ROSA) that endogenously expresses MRGPRX2 at high levels, which we confirmed by flow cytometry (Fig. 4a). Nanobody Sim8619 (rank 1), Sim9877 (rank 5), Sim4784 (rank 7), and Sim4177 (rank 90) showed high levels of binding to ROSA cells, while our negative control nanobody GPCR binder, Nb 60, showed no appreciable binding^24^ (Fig. 4b). Sim8619 (rank 1), Sim9877 (rank 5), and Sim4784 (rank 7) demonstrated reasonably monodispersed size exclusion chromatography profiles and were selected for further validation experiments. However, Sim4177 (rank 90) was poorly behaved biochemically (Supplementary Fig. 4A, B) and was therefore excluded from future analyses. All our candidate nanobodies had a population that eluted in the void during size exclusion chromatography, suggesting that like many nanobodies from synthetic libraries used in yeast or phage display, nanobodies from our in silico screen are generally less well-behaved than nanobodies from a post-immune selection. We further measured candidate nanobody binding to mast cells in dose-response format. Sim8619, Sim9877, and Sim4784, purified as Fc-fusions, bound ROSA mast cells in a dose-dependent manner (Fig. 4c–e). In complementary experiments, HEK293T cells (which lack endogenous MRGPRX2) were transfected with either MRGPRX2 or empty vector. In HEK293T cells, nanobody Sim8619, Sim9877, and Sim4784 showed similar binding properties and estimated dissociation constants as in ROSA mast cells, providing evidence that the nanobodies are binding specifically to MRGPRX2 (Table 1) (Supplementary Fig. 5A–C). We also measured the binding of the nanobodies, expressed in monovalent format, to HEK293T cells expressing MRGPRX2. All the candidate nanobodies showed similar binding properties to their Fc-fusion counterpart (Supplementary Fig. 5E–H). To evaluate the specificity of the nanobodies, we tested the binding of Sim8619, Sim9877, and Sim4784 to two other peptide-binding GPCRs, MC4R and CXCR3 (Supplementary Fig. 5I–K). Sim8619 and Sim9877 showed high specificity for MRGPRX2, while Sim4784 showed some off-target binding to MC4R.

**Table 1:** MRGPRX2 binding characteristics for the three leading candidate nanobodies in HEK293T cells and ROSA mast cells

	HEK293T cells		ROSA mast cells
	Kd (nM ± SEM)	Bmax (Relative ± SEM)	Kd (nM ± SEM)
Nanobody Sim8619 (rank 1)	200 ± 20	97% ± 3	100 ± 10
Nanobody Sim9877 (rank 5)	160 ± 30	100% ± 6	20 ± 4
Nanobody Sim4784 (rank 7)	80 ± 30	43% ± 5	50 ± 10

**Fig. 5:**
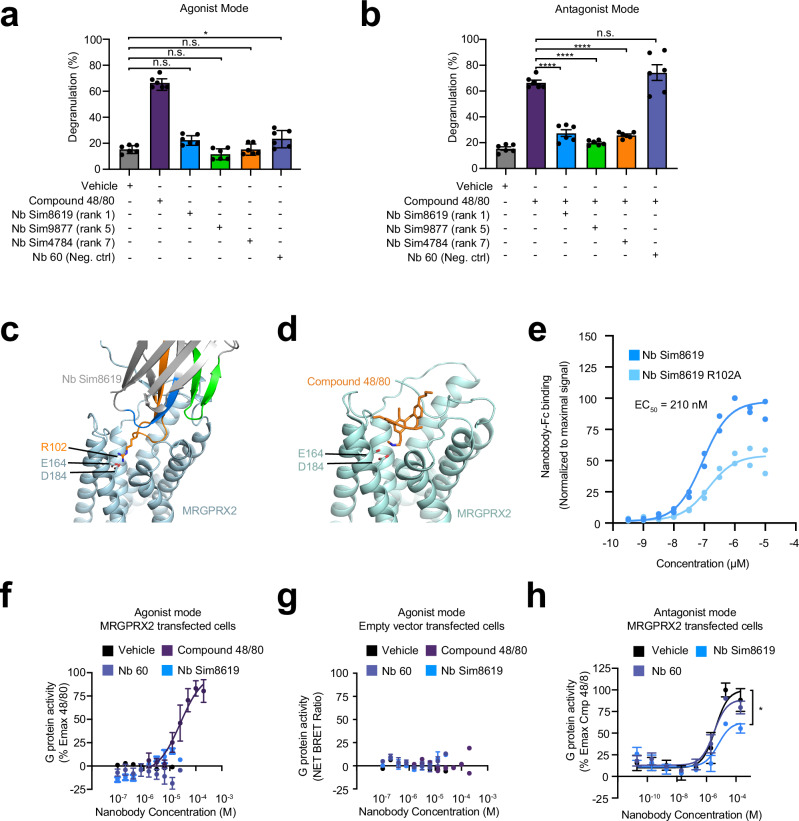
Functional characterization of MRGPRX2 nanobody binders. **a** “Agonist mode” degranulation assay, where degranulation of ROSA cells from Sim8619 (rank 1), Sim9877 (rank 5), and Sim4784 (rank 7) treatment was compared to the positive control agonist Compound 48/80 or the negative control Nb 60. **b** “Antagonist mode” degranulation assay, where ROSA mast cells were pre-treated for 15 min with either Sim8619 (rank 1), Sim9877 (rank 5), and Sim4784 (rank 7), then treated with Compound 48/80 (50 μM). For **a** and **b**, six biological replicates were performed and included at least two separately prepared nanobody purifications. Error bars indicate mean ± SEM of six biological replicates. Statistical analysis was performed using ANOVA, and a Dunnett’s post hoc two-sided test was performed comparing all samples to vehicle treatment ^****^p < 0.0001, ^***^p < 0.0002, ^**^p < 0.0021, ^*^p < 0.0332, n.s. not significant. For **a**, exact *p*-values are: vehicle vs. Compound 48/80: <0.0001; vehicle vs. Nb Sim 8619: 0.1595; vehicle vs. Nb Sim9877: 0.6626; vehicle vs. Nb Sim4177: >0.999; vehicle vs. Nb 60: 0.0146. For **b**, exact *p*-values are: Compound 48/80 vs. Nb Sim 8619, Nb Sim9877, and Nb Sim4177: <0.0001; Compound 48/80 vs. Nb 60: 0.8063. **c** R102 of Sim8619 (rank 1) is predicted in the AF-M structural model to make electrostatic interactions with two acidic residues (E164 and D184) on MRGPRX2. **d** The small molecule agonist Compound 48/80 occupies the same binding site on MRGPRX2 that Sim8619 (rank 1) is predicted to bind, interacting with E164 and D184 (PDB 7VV6). **e** Nanobody Sim8619 (rank 1) with an R102A mutation binds to ROSA mast cells with a weaker EC_50_ (210 nM) and lower maximal binding value compared to wild type Sim8619. Data in **e** represents two biological replicates. G protein activation as assessed by TRUPATH Gi heterotrimer BRET dissociation assay in HEK293T cells overexpressing TRUPATH Gi BRET constructs and MRGPRX2 (**f**) or TRUPATH Gi BRET constructs and empty vector (pcDNA 3.1) (**g**) Sim8619 (rank 1) did not cause appreciable G protein activation relative to the positive control agonist 48/80. **h** HEK293T cells overexpressing TRUPATH Gi BRET constructs and MRGPRX2 were pretreated with Sim8619 (rank 1), negative control antibody  Nb 60, or vehicle for 45 min, and subsequently treated with the indicated concentration of MRGPRX2 agonist Compound 48/80. For **f**, experiments were performed in *n* = 3 biological replicates. For **g**, experiments were performed in *n* = 3 biological replicates, aside from the Compound 48/80 condition, which was performed in biological duplicate. For **h**, experiments were performed in *n* = 4 biological replicates. Error bars indicate mean ± SEM. in all panels. For **h**, ^*^p < 0.05 two-way ANOVA, main effect of nanobody pretreatment. Exact *p*-value: 0.0487. Source data are provided as a Source Data file.

Next, we characterized the functional activity of the three putative MRGPRX2 binders. Activation of MRGPRX2 leads to mast cell degranulation^25,26^, and we used an established β-hexosaminidase release assay to measure the extent of degranulation upon the addition of the nanobodies to mast cells^27^. Known MRGPRX2 small molecule agonist Compound 48/80 induced the release of approximately 70% of granules from mast cells (Fig. 5a). The addition of Sim8619 (rank 1), Sim9877 (rank 5), or Sim4784 (rank 7) did not result in an increase in degranulation over the vehicle or Nb 60 negative control, indicating that these nanobodies are not functional agonists of MRGPRX2 (Fig. 5a). Next, we tested if Sim8619 (rank 1), Sim9877 (rank 5), and Sim4784 (rank 7) were functional antagonists by assessing their ability to block Compound 48/80 mediated degranulation. While the negative control nanobody, Nb 60, had no effect on Compound 48/80 mediated degranulation, the addition of Sim8619 (rank 1), Sim9877 (rank 5), or Sim4784 (rank 7) attenuated Compound 48/80-induced degranulation, suggesting they are functional antagonists (Fig. 5b).

Interestingly, AF-M predicts Sim8619 (rank 1), Sim9877 (rank 5), and Sim4784 (rank 7) bind in the same orthosteric pocket site as Compound 48/80 (Supplementary Fig. 6A–D). The CDR3s of nanobody Sim8619 (rank 1) and nanobody Sim 4784 (rank 7) mimic the binding interaction of a positively charged side group of the MRGPRX2 agonist Compound 48/80 with two acidic residues, E164 and D184, on the receptor (Fig. 5c, d, and Supplementary Fig. 6A–D), which have previously been shown to be essential for Compound 48/80’s agonist activity^28–30^. To test this predicted binding pose, we mutated a critical arginine residue (R102A) within our top-ranked nanobody, Sim8619 (rank 1), which the AF-M predicted alignment suggested would disrupt binding with E164 and D184 in the MRGPRX2 orthosteric site (Fig. 5c, d). In support of this binding pose, the R102A mutant bound to ROSA cells with a twofold decreased affinity and lower maximal binding (Fig. 5e). The predicted similarity of these binding interactions provides a structural rationale for the ability of Sim8169 to block Compound 48/80 mediated degranulation. To further validate the predicted binding poses of Sim8619 (rank 1), Sim9877 (rank 5), and Sim4784 (rank 7), we created a panel of nanobody constructs with mutations in their CDRs that are predicted to disrupt interaction with MRGPRX2 in the AlphaFold models. Like the R102A mutation, these additional mutations decreased affinity, suggesting that AlphaFold can accurately predict their binding poses (Supplementary Fig. 6e–g, and Supplementary Table 4). To provide orthogonal validation of the binding poses of the nanobodies, we mutated the previously mentioned two acidic residues in MRGPRX2 (E164 and D184) and measured the effects of these mutations on nanobody binding. While Sim8619 (rank 1) and Sim4784 (rank 7) both contain an arginine residue within their CDR3 that is predicted by the AF-M model to form a salt bridge with E164/D184, residues within the CDR3 of Sim9877 (rank 7) are not predicted to make significant interactions with E164/D184 **(**Supplementary Fig. 6B–D). In line with this, Sim9877 (rank 5) did not show a significant change in binding to mutant MRGPRX2, while Sim8619 (rank 1) and Sim4784 (rank 7) both exhibited a significant reduction in binding compared to wild type MRGPRX2 (Supplementary Fig. 6H–J).

Based on these predictions and binding data, we proceeded to test if Sim8169 (rank 1) could directly compete with the MRGPRX2 agonist Compound 48/80 in its ability to activate the G protein Gi, an established signaling output of MRGPRX2^31^. We first tested the ability of Sim8619 to independently activate Gi. Unlike Compound 48/80, which robustly activated Gi in an MRGPRX2-dependent manner, Sim8619 did not, with only slight G protein activity only at high concentrations relative to the agonist compound 48/80 (Fig. 5f, g). However, pretreatment of MRGPRX2-expressing cells with Sim8619 prior to Compound 48/80 treatment lowered the G protein activity Emax and slightly right-shifted the EC_50_ (Fig. 5h). To further interrogate Sim8619’s mode of antagonism, we pre-treated MRGPRX2-expressing cells with Sim8619 prior to treatment with Substance P, a structurally dissimilar MRGPRX2 peptide agonist that binds within the same orthosteric pocket. Again, we observed that the G protein activity Emax was reduced and the EC_50_ was right-shifted (Supplementary Fig. 5L). These competition assays provide further support for the predicted binding site of Sim8169 in MRGPRX2, as well as support for Sim8169 acting as a functional MRGPRX2 antagonist.

## Discussion

Small-molecule docking is widely used to identify compounds that bind to proteins of interest. In silico compound docking helps make laboratory work more efficient by enriching for compound binders rather than naively screening chemical space. Similarly, here we show that AF-M can be deployed to screen GPCR-binding nanobodies by ranking nanobody-GPCR interactions according to their AF-M confidence metrics. Our results are in line with other recent benchmarking studies which evaluate the ability of AlphaFold-Multimer to predict the structures of nanobodies bound to antigens^32,33^. However, we specifically show here that AlphaFold-Multimer is uniquely adept at predicting nanobodies bound to GPCRs compared to other classes of antigens and can be used in prospective screening for nanobody binders to GPCRs. We show that AF-M can accurately identify true nanobody binders to the GPCR MRGPRX2. We identified three nanobody binders to MRGPRX2 and validated that these nanobodies have functional antagonist activity. Despite the improved capabilities of AF-M over its predecessors, these findings are in some ways surprising, as unlike many other protein-protein interactions, co-evolution between nanobodies or antibodies and GPCRs does not occur. This lack of co-evolution between antibodies and their binding partners limits the utility of one of the core metrics used in AF-M predictions. In recent years, the number of structures of nanobodies bound to GPCRs has increased rapidly, increasing from a handful in 2015 to more than 150 deposited structures in 2023 (Fig. 1c). This rapid increase in reliable training data may, in part, explain our success with a GPCR and not with other protein targets, as AF-M has more examples within its training set to utilize. Future iterations of AF-M or similar models are likely to continue to improve the ability to recognize antibody binders accurately.

We chose MRGPRX2 as a test candidate because of its clinical potential and because it is a known promiscuous binder, thereby making small library screening more likely to succeed. The shallow binding pocket accommodates a broad range of charged ligands^34^. Further studies are needed to determine if this strategy can be deployed similarly for other GPCRs. In our study, nanobody Sim8619 (rank 1), nanobody Sim9877 (rank 5), and nanobody Sim4784 (rank 7) are nanomolar binders and function as MRGPRX2 antagonists, as assessed by blocking mast cell degranulation. Derivatives of these nanobodies could be of therapeutic interest for diseases such as chronic spontaneous urticaria^35^. A recent study reported the discovery of MRGPRX1 agonist mini-proteins in which tens of thousands of candidate binders were designed to bind the active state of MRGPRX1 and were screened in a high-throughput cell binding assay^36^. In contrast, we report the identification of functional antagonist molecules discovered through an AF-M screen against the inactive state of MRGRX2 and recombinant expression and purification of a modest set of ten nanobodies that possessed the highest AF-M confidence metrics. Importantly, while extensive mutagenesis was used to validate the predicted binding poses of our nanobodies, we experienced technical difficulties purifying sufficient amounts of properly folded MRGPRX2, precluding structural work. The precise mode of antagonism and the binding pose of the nanobodies remain open questions in the absence of experimental structures.

More broadly, antibodies have emerged as essential reagents for deciphering GPCR biology. Approximately 35% of FDA-approved drugs target GPCRs, highlighting that pharmacologic modulation of GPCRs is a critical cornerstone of modern medicine^37^. Antibodies can offer certain advantages over small molecules targeting GPCRs in many cases, such as extended half-life and often increased specificity. However, as of 2021, few GPCR-targeting antibodies are currently in clinical development, with only three GPCR-targeting monoclonal antibodies approved, and less than 1% of the GPCR therapeutic pipeline consists of antibodies^38^. This deficit is in large part due to the technical challenges associated with conducting GPCR antibody discovery campaigns. GPCRs are conformationally dynamic and often poorly expressed, making it particularly challenging to purify the quantities of biochemically stable and homogenous samples needed for traditional antibody discovery campaigns.

Our proof-of-concept demonstration that AF-M can be used in prospective screening to identify MRGPRX2 nanobody binders raises an important and exciting question: can similar AF-M-based approaches be used to find nanobody binders to other GPCRs or receptor types? Indeed, the field of antibody and protein design is rapidly progressing, enabled by advances in artificial intelligence. Chai-2, a multimodal generative model for structure prediction and design, was recently reported to have success in identifying nanobodies to several GPCRs in silico, including functional agonists^39^. JAM was also deployed to generate in silico VHHs for multi-pass membrane proteins including a GPCR^40^. Likewise, RFdiffusion, BoltzGen, Germinal, and mBER have reported success in identifying nanobody binders for several target types^14,41,42^. For AF-M-based screening methods, we expect that larger in silico libraries will be needed to successfully find binders to other GPCRs, which will be computationally challenging. For more challenging in silico targets, Bayesian optimization methods such as active learning can be deployed to learn the binding contributions of specific amino acids from a subset of antibodies screened to guide the design of remaining library members and more efficiently explore antibody chemical space^43^. Additionally, in silico libraries could be designed around sequences previously identified to bind to GPCRs. As more structures of antibodies bound to proteins continue to be deposited in the PDB, the predictive ability of AF-M and other computational models, such as RFdiffusion, will likely increase, further improving the already noteworthy utility of this method to discover new antibody fragment binders and decipher other protein-protein interactions.

## Methods

### AF-M and AlphaFold3 multimer predictions

AF-M structural predictions were generated using a modified local ColabFold script (https://github.com/YoshitakaMo/localcolabfold) operated on a Lambda Labs server with a NVIDIA A100 GPU^8,10^. In particular, AF-M multimer v2 or v3 was used with three recycles and was run without templates and amber processing^12^. PyMol was used to visualize AF-M multimer structural predictions, and confidence in predictions was assessed by contact analysis scripts. AlphaFold3 structural predictions were generated using the AlphaFold3 server (http://alphafoldserver.com) with standard settings, and ipTM/pTM values were obtained from the server output^9^.

### ESMFold multimer predictions

ESMFold binding complex structure predictions were generated using standard run scripts with the multimer setting (https://github.com/facebookresearch/esm/tree/main#esmfold)^19^. Models were run using a single NVIDA Tesla V100S GPU. Predicted structures contained predicted-LDDTs (pLDDTS) which were parsed from the output.pdb file. Average pLDDTs of the residues in the full VHH, all the CDR regions, and just CDR3 were used as binding scores from ESMFold. To retrieve the CDR annotations, IMGT numbering of the nanobody sequences in the folding benchmark was performed using ANARCI^44^.

### Recombinant nanobody-Fc fusion expression and purification

To recombinantly express nanobodies fused to human IgG1 Fc, nanobody DNA sequences followed by a (GGS)3 linker were cloned into pFUSE-CHIg-hG1 (InVivo Gen) containing a H435A substitution, which prevents IgG Fc binding to Protein A resin^45,46^. Expi293F cells (Thermo Fisher Scientific, Cat No: A14635) were then transiently transfected with nanobody plasmids. Briefly, 200 mL of Expi293F cells cultured in Expi293 expression media (Thermo-Fisher) were grown to a density of 3 × 10^6^ cells/mL. Then, cells were transiently transfected using nanobody DNA (0.16 mg total DNA) and FectoPro transfection reagent (Polyplus) at a 1:1 DNA/FectoPro ratio. 16 h after transfection, cells were enhanced with 3 mM Valproic acid sodium salt (Sigma-Aldrich) and 0.8% D-(+)-Glucose (Sigma-Aldrich). Transfected cells were cultured for 6 days to produce nanobody Fc-fusions. Then, the media was separated from cells by centrifugation at 4000xg for 15 min at 4 °C, and applied to protein A resin equilibrated with 20 mM HEPES, 150 mM NaCl (pH 7.5). The protein A resin was then washed with 20 column volumes 20 mM HEPES, 150 mM NaCl (pH 7.5). Following resin washing, nanobody IgG1 Fc fusions were eluted from the resin using 100 mM citrate (pH 3) directly into 2 M HEPES (pH 8) and the pH of the eluted and neutralized protein solution was checked using pH strips. Eluted nanobody Fc fusions were then dialyzed overnight in 20 mM HEPES, 150 mM NaCl, 10% glycerol (pH 7.5), and were flash frozen. Protein purity was assessed by SDS-PAGE and analytical size exclusion chromatography runs on a Superdex 200 Increase 3.2/300 gel filtration column (GE Healthcare).

### Recombinant monomeric nanobody expression and purification

Monomeric nanobodies and nanobody variants with C-terminal V5 and hexa-histadine tags were expressed via a pET-26b expression vector in BL21-DE3 *Escherichia coli* from 250 mL cultures and purified using a high-throughput modified protein A and nickel affinity workflow. Following expression, bacterial pellets were harvested by centrifugation (multiple spins as required) and stored at –20 °C until use. Cell pellets were resuspended in 10 mL of SET lysis buffer (200 mM Tris, pH 8.0, 500 mM sucrose, 500 µM EDTA) and incubated at room temperature with gentle rotation. After addition of 20 mL ice-cold water, 5 mM MgCl₂, and 0.5 µL benzonase nuclease, lysates were rotated for 1 h at room temperature and centrifuged at 14,000 × *g* for 20–30 min. Supernatants transferred to fresh 50 mL conical tubes and were supplemented with 100 mM NaCl and incubated with 2 mL Protein A resin (equilibrated as a 50% slurry in wash buffer) for 1 h at room temperature with gentle agitation to select for appropriately folded nanobodies. Protein A resin was pelleted (500 × *g*, 3–5 min) and washed with 10 column volumes of wash buffer with intermittent mixing, then eluted in 8–10 mL of 100 mM NaH_2_PO_4_ 100 mM NaCl, pH 2.5 for 20–30 min with gentle agitation. Tubes were spun down, and elutant transferred to tubes preloaded with 5 mL of 2 M HEPES. and once more centrifuged (4000 × *g*, 2–3 min) to remove residual resin. The Protein A–purified material was then batch-incubated overnight at 4 °C with 2 mL Ni–NTA resin (pre-equilibrated in HBS buffer: 20 mM HEPES pH 7.5, 150 mM NaCl). The following day, resin–nanobody mixtures were transferred to gravity-flow columns. Columns were washed sequentially with 10 CV of Wash Buffer 1 (20 mM HEPES pH 7.5, 500 mM NaCl, 20 mM imidazole) and 10 CV of Wash Buffer 2 (20 mM HEPES pH 7.5, 100 mM NaCl, 20 mM imidazole). Bound nanobodies were eluted with 8–10 mL Elution Buffer (20 mM HEPES pH 7.5, 100 mM NaCl, 200 mM imidazole).

Eluted proteins were dialyzed overnight at 4 °C into size-exclusion/dialysis buffer (20 mM HEPES pH 7.5, 150 mM NaCl, 10% glycerol). Nanobodies were then concentrated to >100 µM (typically ~1.6 mg/mL) using 10 kDa cutoff centrifugal concentrators (Amicon). Final protein preparations were aliquoted and flash frozen. Protein integrity and purity were confirmed by SDS–PAGE.

### G protein signaling assays

The Gi TRUPATH bioluminescence energy transfer (BRET) integrated plasmid was a gift from Justin English. Assays were conducted similar to those previously described^47–49^. Briefly, HEK293T cells (ATCC: CRL-3216) were transfected with a human N-terminal FLAG-tagged MRGPRX2 in a pcDNA3.1 expression vector or empty vector control and a Gi TRUPATH plasmid. After 24 h, cells were plated in a 96-well plate in phenol red-free DMEM supplemented with 2% FBS, 1% Glutamax, and 1% antibiotic-antimycotic (Sigma). Approximately 48 h after transfection, media was replaced with assay buffer (HBSS without calcium or magnesium + 20 mM HEPES + 3 µM coelenterazine-400a (Nanolight) and BRET ratios were obtained using a Promega plate reader. For agonist mode, data were normalized to a pre-read prior to nanobody treatment. For the initial MRGPRX2 activation screen, cells were treated with 20 µM of each MRGPRX2 simulation nanobody and read approximately 10 min after addition. For competition assay (“antagonist mode”), cells were pretreated with 10 µM of the indicated nanobody or vehicle for 45 min, and then a pre-read was conducted. Cells were then treated with variable concentrations of the MRGPRX2 agonist Compound 48/80, Substance P, or vehicle, and the net BRET ratio was calculated by first subtracting the pre-read from the post-read, then subtracting vehicle pre-treatment, vehicle treatment condition from other treatment conditions. Independent experiments were conducted in technical triplicate and merged for a biological replicate, with the indicated number of biological replicates per experiment included within the pertinent figure legends.

### Nanobody-Fc fusion on-cell cytometry binding assays

The wild type ROSA mast cell line^50^ was a gift from the Galli lab (Stanford University) and was confirmed negative for mycoplasma. ROSA cells were cultured at 37 °C and 5% CO_2_ in IMDM supplemented with 1% Penicillin-Streptomycin (Pen-Strep), 1% sodium pyruvate (GIBCO), 1% MEM (minimal essential medium) (GIBCO), 2% MEM non-essential amino acids (GIBCO), 1% L-glutamine (GIBCO), 1% insulin transferrin-sodium selenite (GIBCO), 0.3% bovine serum albumin, with 80 ng/mL fresh mouse stem cell factor (SCF) (R&D Systems). To obtain nanobody dose response curves, cells were harvested by centrifugation at 1000 × *g* and washed twice with cold phosphate buffered saline (PBS). The cells were then counted, and 100,000 cells were added to each well of a V-bottom 96 well plate (Grenier Bio). Cells were washed again with 100 μL cold PBS and then were incubated with different concentrations of either the candidate nanobodies, commercial MRGPRX2 antibody (Biolegend Cat# 359005, AB_2750139), or the negative control Nb 60-Fc fusion (β2AR internal nanobody binder negative control), or buffer alone for 30  min at 4 °C^24^. Following another PBS wash, cells were incubated with 1 μg/mL anti-human IgG Fc-AF488 (Invitrogen Cat#A-11013) for 30 min at 4 °C. After secondary antibody incubation, cells were washed twice more with PBS and were analyzed by flow cytometry using a CytoFLEX cytometer (Beckman-Coulter). In detail, live/dead gates were set using SSC-A versus FSC-A and singlet gates using FSC-H versus FSC-A, and fluorescence intensity at 488 nm was recorded in the doubly gated population. Mean fluorescence intensity (MFI) at 488 nm was plotted in GraphPad Prism as an assessment of nanobody binding to cells. For Kd calculations, non-specific signal was MFI in the Nb 60 condition.

For HEK293 binding assays, HEK293T cells were cultured at 37 °C and 5% CO_2_ in DMEM supplemented with 10% FBS and 1% Pen-Strep. Cells were seeded into a six well plate and at ~50% confluency were transiently transfected using Fugene (Promega) with 500 ng of N-terminal FLAG-tagged MRGPRX2 or MRGPRX2 E164A D184A expression plasmid or empty vector pcDNA negative control, similar to previously described^48^. 48 h later, cells were removed from the plate with PBS, pelleted, and resuspended in HBS supplemented with 2% FBS and 0.05% bovine serum albumin (BSA). ~100,000 cells were seeded into a 96-well plate, pelleted, and treated with the indicated concentration of nanobody-Fc for 1 h or vehicle at 4 °C in duplicate, shaking at 30 rpm. Cells were pelleted and washed with HBS + 2% FBS + 0.05% BSA, then treated with anti-human Fc-AF647 (Biolegend Cat. #410714) for 20 min. Cells were pelleted and washed with HBS + 2% FBS + 0.05% BSA, and resuspended in HBS + 2% FBS + 0.05% BSA + 0.4% formaldehyde. Cells were analyzed by cytometry using a CytoFLEX cytometer (Beckman-Coulter), and gated by forward-scatter/side scatter, and singlets (FSC-A versus FSC-H). Mean fluorescence intensity (MFI) at 647 nm within this singlet population was used as the assessment for nanobody binding. Experiments were conducted on two separate days in technical duplicate, and technical duplicates were averaged for each replicate. Nanobodies were tested simultaneously to allow for Bmax and Kd comparisons. Maximal signal was normalized to each 1 μM nanobody-Fc condition. For Kd and Bmax calculations, non-specific signal was MFI at the indicated concentration in HEK293 cells transfected with the empty vector negative control. Data were analyzed in GraphPad Prism version 10.

### Monomeric nanobody on-cell cytometry binding assays

To assess nanobody binding specificity, HEK293T cells were transiently transfected with plasmids encoding empty vector, FLAG-MRGPRX2, FLAG-MC4R, or FLAG-CXCR3 as described above. For flow cytometry experiments, cells were washed in PBS, pelleted (500 × *g*, 5 min, 4 °C), and washed once with HBS supplemented with 0.1% BSA (w/v) + 2 mM calcium (flow buffer). Cells were resuspended in flow buffer and distributed into 96-well plates (100 µL per well). Following centrifugation (500 × *g*, 5 min), supernatants were removed, and cells were resuspended in 100 µL staining buffer containing monomeric nanobody or monomeric nanobody variant with a V5 tag and incubating for 1–2 h at 4 °C with gentle agitation (30 rpm). For peptide comparison experiments, this was conducted at a single high saturating concentration of monomeric nanobody (1–2 µM). After two washes with 150–200 µL flow buffer, cells were incubated for 20–30 min at 4 °C with 100 µL of secondary detection reagent anti-V5 antibody conjugated to APC (made in house) diluted in flow buffer. Cells were washed once, then flow cytometry was either immediately performed after resuspension in 100 µL of flow buffer, or cells were fixed in 100 µL of flow buffer containing 0.4% paraformaldehyde and flow cytometry was performed the following day. Data were analyzed by gating on forward-scatter area (FSC-A), side-scatter area (SSC-A), and singlets.

### β-hexosaminidase release assay

To measure β-hexosaminidase release, ROSA cells were washed twice in warm (37 °C) Tyrode’s buffer (20 mM HEPES with 134 mM NaCl, 5 mM KCl, 1.8 mM CaCl_2_, 1 mM MgCl_2_, 5.5 mM glucose, and 0.3% bovine serum albumin, pH 7.4). Cells were then seeded at 100,000 cells per well (100 μL, 1x10^6^ cells/mL) in a flat bottom clear 96-well plate. Cells were pre-treated with nanobody by adding nanobody at 2× working concentration (100 µM) in Tyrode’s buffer and then cells were incubated for 15 min in a humidified incubator with 5% CO_2_ at 37 °C. After the 15 min incubation, Compound 48/80 (Sigma) was added at 50 µM final concentration or nanobody at 50 µM final concentration, with final volumes for all conditions kept at 200 µL. Cells were incubated for 60 min in a humidified incubator with 5% CO_2_ at 37 °C. After incubation, samples were transferred to a v-bottom plate, and plates were centrifuged at 500 × *g* for 5 min. For the supernatant-only reading, 50 μL supernatant was added to a new 96-well black flat-bottom plate (Nunc) containing 50 μL of 5 μM 4-Methylumbelliferyl-β-D-glucopyranosiduronic acid (4 MUG) (Sigma) diluted in 100 mM citrate buffer (pH 4.5). For the supernatant and lysate reading, cells were lysed by adding 20 μL of 10% Triton-X-100 to each well (0.01% Triton-X-100 final). Samples were mixed vigorously with pipetting to break open the cells. 50 μL of lysate was added to 50 μL of 5 μM 4 MUG. All plates were incubated for 60 min in an incubator without CO_2_ at 37 °C. Lastly, 100 µL of 400 mM glycine pH 10.7 was added to each well to stop the reaction. Fluorescence intensity at 360/460 was read on a Clariostar (BMG LabTech) with the top optic. Percent degranulation was calculated using the formula: % degranulation= ((supernatant)/(supernatant+lysate))×100.

### Statistical methods

Prism software (Graphpad) was used to analyze data and perform error calculations. Data are expressed as arithmetic mean ± SEM.

### Reporting summary

Further information on research design is available in the Nature Portfolio Reporting Summary linked to this article.

## Supplementary information

Supplementary Information

Description of Additional Supplementary Files

Supplementary Data 1

Supplementary Data 2

Supplementary Data 3

Supplementary Data 4

Supplementary Data 5

Reporting Summary

Transparent Peer Review file

## Source data

Source Data

## Data Availability

The data that support this study are available from the corresponding authors upon request. Source data are provided with this paper.

## Code availability

Code for analyzing AF-M results and ranking nanobody clones can be found on GitHub: https://github.com/kruselab/MRGPRX2-AF-M-screen.

## References

[1] Carter, P. J. & Rajpal, A. Designing antibodies as therapeutics. *Cell* **185**, 2789–2805 (2022). 35868279 10.1016/j.cell.2022.05.029

[2] Banik, S. S. R., Kushnir, N., Doranz, B. J. & Chambers, R. Breaking barriers in antibody discovery: harnessing divergent species for accessing difficult and conserved drug targets. *mAbs* **15**, 2273018 (2023). 38050985 10.1080/19420862.2023.2273018 PMC10793698

[3] Sharma, P., Joshi, R. V., Pritchard, R., Xu, K. & Eicher, M. A. Therapeutic antibodies in medicine. *Molecules* **28**10.3390/molecules28186438 (2023). 10.3390/molecules28186438 PMC10535987 37764213

[4] Morrison, C. Nanobody approval gives domain antibodies a boost. *Nat. Rev. Drug Discov.* **18**, 485–487 (2019). 31267082 10.1038/d41573-019-00104-w

[5] De Pauw, T. et al. Current status and future expectations of nanobodies in oncology trials. *Expert Opin. Investig. Drugs* **32**, 705–721 (2023). 37638538 10.1080/13543784.2023.2249814

[6] Lu, R. M. et al. Development of therapeutic antibodies for the treatment of diseases. *J. Biomed. Sci.* **27**10.1186/s12929-019-0592-z (2020). 10.1186/s12929-019-0592-z PMC6939334 31894001

[7] Harvey, E. P. et al. An in silico method to assess antibody fragment polyreactivity. *Nat. Commun.* **13**, 7554 (2022). 36477674 10.1038/s41467-022-35276-4 PMC9729196

[8] Mirdita, M. et al. ColabFold: making protein folding accessible to all. *Nat. Methods* **19**, 679–682 (2022). 35637307 10.1038/s41592-022-01488-1 PMC9184281

[9] Abramson, J. et al. Accurate structure prediction of biomolecular interactions with AlphaFold 3. *Nature*10.1038/s41586-024-07487-w (2024). 10.1038/s41586-024-07487-w PMC11168924 38718835

[10] Jumper, J. et al. Highly accurate protein structure prediction with AlphaFold. *Nature* **596**, 583–589 (2021). 34265844 10.1038/s41586-021-03819-2 PMC8371605

[11] Senior, A. W. et al. Improved protein structure prediction using potentials from deep learning. *Nature* **577**, 706–710 (2020). 31942072 10.1038/s41586-019-1923-7

[12] Evans, R. et al. Protein complex prediction with AlphaFold-Multimer. Preprint at *bioRxiv* https://www.biorxiv.org/content/10.1101/2021.10.04.463034v2 (2022).

[13] Watson, J. L. et al. De novo design of protein structure and function with RFdiffusion. *Nature* **620**, 1089–1100 (2023). 37433327 10.1038/s41586-023-06415-8 PMC10468394

[14] Bennett, N. R. et al. Atomically accurate de novo design of single-domain antibodies. Preprint at *bioRxiv*10.1101/2024.03.14.585103 (2024).

[15] Skiba, M. A. et al. Antibodies expand the scope of angiotensin receptor pharmacology. *Nat. Chem. Biol*. 10.1038/s41589-024-01620-6 (2024). 10.1038/s41589-024-01620-6 PMC11561159 38744986

[16] Schmid, E. W. & Walter, J. C. Predictomes: a classifier-curated database of AlphaFold-modeled protein-protein interactions. *Mol Cell.* **85**, 1216–1232.e5 (2025). 10.1016/j.molcel.2025.01.034 PMC11931459 40015271

[17] Lim, Y. et al. In silico protein interaction screening uncovers DONSON’s role in replication initiation. *Science* **381**, eadi3448 (2023). 37590370 10.1126/science.adi3448 PMC10801813

[18] Basu, S. & Wallner, B. DockQ: a quality measure for protein-protein docking models. *PLoS ONE* **11**, e0161879 (2016). 27560519 10.1371/journal.pone.0161879 PMC4999177

[19] Lin, Z. et al. Evolutionary-scale prediction of atomic-level protein structure with a language model. *Science* **379**, 1123–1130 (2023). 36927031 10.1126/science.ade2574

[20] McMahon, C. et al. Yeast surface display platform for rapid discovery of conformationally selective nanobodies. *Nat. Struct. Mol. Biol.* **25**, 289–296 (2018). 29434346 10.1038/s41594-018-0028-6 PMC5839991

[21] Cao, C. & Roth, B. L. The structure, function, and pharmacology of MRGPRs. *Trends Pharm. Sci.* **44**, 237–251 (2023). 36870785 10.1016/j.tips.2023.02.002 PMC10066734

[22] Cao, C. et al. Structure, function and pharmacology of human itch GPCRs. *Nature* **600**, 170–175 (2021). 34789874 10.1038/s41586-021-04126-6 PMC9150435

[23] Yang, F. et al. Structure, function and pharmacology of human itch receptor complexes. *Nature* **600**, 164–169 (2021). 34789875 10.1038/s41586-021-04077-y

[24] Staus, D. P. et al. Allosteric nanobodies reveal the dynamic range and diverse mechanisms of G-protein-coupled receptor activation. *Nature* **535**, 448–452 (2016). 27409812 10.1038/nature18636 PMC4961583

[25] McNeil, B. D. et al. Identification of a mast-cell-specific receptor crucial for pseudo-allergic drug reactions. *Nature* **519**, 237–241 (2015). 25517090 10.1038/nature14022 PMC4359082

[26] Azimi, E. et al. Dual action of neurokinin-1 antagonists on Mas-related GPCRs. *JCI Insight* **1**, e89362 (2016). 27734033 10.1172/jci.insight.89362 PMC5053144

[27] Kuehn, H. S., Radinger, M. & Gilfillan, A. M. Measuring mast cell mediator release. *Curr. Protoc. Immunol.*10.1002/0471142735.im0738s91 (2010). 10.1002/0471142735.im0738s91 PMC2982193 21053305

[28] Reddy, V. B., Graham, T. A., Azimi, E. & Lerner, E. A. A single amino acid in MRGPRX2 necessary for binding and activation by pruritogens. *J. Allergy Clin. Immunol.* **140**, 1726–1728 (2017). 28689793 10.1016/j.jaci.2017.05.046 PMC5723232

[29] Lansu, K. et al. In silico design of novel probes for the atypical opioid receptor MRGPRX2. *Nat. Chem. Biol.* **13**, 529–536 (2017). 28288109 10.1038/nchembio.2334 PMC5391270

[30] Alkanfari, I., Gupta, K., Jahan, T. & Ali, H. Naturally occurring missense MRGPRX2 variants display loss of function phenotype for mast cell degranulation in response to substance P, hemokinin-1, human beta-defensin-3, and icatibant. *J. Immunol.* **201**, 343–349 (2018). 29794017 10.4049/jimmunol.1701793 PMC6039248

[31] Tatemoto, K. et al. Immunoglobulin E-independent activation of mast cell is mediated by Mrg receptors. *Biochem. Biophys. Res. Commun.* **349**, 1322–1328 (2006). 16979137 10.1016/j.bbrc.2006.08.177

[32] Yin, R. & Pierce, B. G. Evaluation of AlphaFold antibody-antigen modeling with implications for improving predictive accuracy. *Protein Sci.* **33**, e4865 (2024). 38073135 10.1002/pro.4865 PMC10751731

[33] Eshak, F. & Goupil-Lamy, A. Advancements in nanobody epitope prediction: a comparative study of AlphaFold2Multimer vs AlphaFold3. *J. Chem. Inf. Model* **65**, 1782–1797 (2025). 39927847 10.1021/acs.jcim.4c01877

[34] Al Hamwi, G. et al. MAS-related G protein-coupled receptors X (MRGPRX): Orphan GPCRs with potential as targets for future drugs. *Pharm. Ther.* **238**, 108259 (2022). 10.1016/j.pharmthera.2022.108259 35934214

[35] Shtessel, M. et al. MRGPRX2 activation causes increased skin reactivity in patients with chronic spontaneous urticaria. *J. Invest. Dermatol.* **141**, 678–681 e672 (2021). 32771471 10.1016/j.jid.2020.06.030 PMC11658616

[36] Muratspahic, E. et al. *De novo* design of miniprotein agonists and antagonists targeting G protein-coupled receptors. Preprint at *bioRxiv*10.1101/2025.03.23.644666 (2025).

[37] Hauser, A. S., Attwood, M. M., Rask-Andersen, M., Schioth, H. B. & Gloriam, D. E. Trends in GPCR drug discovery: new agents, targets and indications. *Nat. Rev. Drug Discov.* **16**, 829–842 (2017). 29075003 10.1038/nrd.2017.178 PMC6882681

[38] Yang, D. et al. G protein-coupled receptors: structure- and function-based drug discovery. *Signal Transduct. Target Ther.* **6**, 7 (2021). 33414387 10.1038/s41392-020-00435-w PMC7790836

[39] Team, C. D. et al. Drug-like antibody design against challenging targets with atomic precision. Preprint at *bioRxiv*10.1101/2025.11.29.691346 (2025).

[40] Bio, N. & Biswas, S. *De novo* design of epitope-specific antibodies against soluble and multipass membrane proteins with high specificity, developability, and function. Preprint at *bioRxiv*10.1101/2025.01.21.633066 (2025).

[41] Mille-Fragoso, L. S. et al. Efficient generation of epitope-targeted de novo antibodies with Germinal. Preprint at *bioRxiv*10.1101/2025.09.19.677421 (2025).

[42] Stark, H. et al. BoltzGen: toward universal binder design. Preprint at *bioRxiv*10.1101/2025.11.20.689494 (2025).

[43] Graff, D. E., Shakhnovich, E. I. & Coley, C. W. Accelerating high-throughput virtual screening through molecular pool-based active learning. *Chem. Sci.* **12**, 7866–7881 (2021). 34168840 10.1039/d0sc06805e PMC8188596

[44] Dunbar, J. & Deane, C. M. ANARCI: antigen receptor numbering and receptor classification. *Bioinformatics* **32**, 298–300 (2016). 26424857 10.1093/bioinformatics/btv552 PMC4708101

[45] Skiba, M. A. Antibodies expand the scope of angiotensin receptor pharmacology. *Nat Chem Biol.* **20**, 1577–1585 (2024). 10.1038/s41589-024-01620-6 PMC11561159 38744986

[46] Martin, W. L., West, A. P. Jr, Gan, L. & Bjorkman, P. J. Crystal structure at 2.8 A of an FcRn/heterodimeric Fc complex: mechanism of pH-dependent binding. *Mol. Cell* **7**, 867–877 (2001). 11336709 10.1016/s1097-2765(01)00230-1

[47] Smith, J. S. et al. The M3 muscarinic acetylcholine receptor can signal through multiple G protein families. *Mol. Pharm.* **105**, 386–394 (2024). 10.1124/molpharm.123.000818 PMC11114115 38641412

[48] Eiger, D. S. et al. Phosphorylation barcodes direct biased chemokine signaling at CXCR3. *Cell Chem. Biol.* **30**, 362–382 e368 (2023). 37030291 10.1016/j.chembiol.2023.03.006 PMC10147449

[49] Olsen, R. H. J. et al. TRUPATH, an open-source biosensor platform for interrogating the GPCR transducerome. *Nat. Chem. Biol.* **16**, 841–849 (2020). 32367019 10.1038/s41589-020-0535-8 PMC7648517

[50] Saleh, R. et al. A new human mast cell line expressing a functional IgE receptor converts to tumorigenic growth by KIT D816V transfection. *Blood* **124**, 111–120 (2014). 24677542 10.1182/blood-2013-10-534685

