# Supplementary material for: In silico discovery of nanobody binders to a G-protein coupled receptor using AlphaFold-Multimer
Source: Nat Commun. 2026 Apr 23;17:5641. doi: 10.1038/s41467-026-72093-5 (PMC13315262; doi:10.1038/s41467-026-72093-5)
Supplement: Supplementary file 1 — Supplementary Information [file 41467_2026_72093_MOESM1_ESM.pdf]

Supplementary Table 1. Nanobody Sequences. CDRs are shown in red.

**Name**	**Sequence** (Bold = mutation from corresponding parent, red italics = CDR regions)
Sim8619 (MRGPRX2 Rank 1)	QVQLQESGGGLVQAGGSLRLSCAASGSIFYIRGMGWYRQAPGKERELVAGIDVGAITYYADSVKGRFTISRDNAKNTVYLQMNSLKPEDTAVYYCAVWAYTRAGYTTVYAYWGQGTQVTVSS
Sim8619 Y100G	QVQLQESGGGLVQAGGSLRLSCAASGSIFYIRGMGWYRQAPGKERELVAGIDVGAITYYADSVKGRFTISRDNAKNTVYLQMNSLKPEDTAVYYCAVWA**G**TRAGYTTVYAYWGQGTQVTVSS
Sim8619 R102A	QVQLQESGGGLVQAGGSLRLSCAASGSIFYIRGMGWYRQAPGKERELVAGIDVGAITYYADSVKGRFTISRDNAKNTVYLQMNSLKPEDTAVYYCAVWAYT**A**AGYTTVYAYWGQGTQVTVSS
Sim8619 Y105D	QVQLQESGGGLVQAGGSLRLSCAASGSIFYIRGMGWYRQAPGKERELVAGIDVGAITYYADSVKGRFTISRDNAKNTVYLQMNSLKPEDTAVYYCAVWAYTRAG**D**TTVYAYWGQGTQVTVSS
Sim8619 Y105G	QVQLQESGGGLVQAGGSLRLSCAASGSIFYIRGMGWYRQAPGKERELVAGIDVGAITYYADSVKGRFTISRDNAKNTVYLQMNSLKPEDTAVYYCAVWAYTRAG**G**TTVYAYWGQGTQVTVSS
Sim7252 (MRGPRX2 Rank 2)	QVQLQESGGGLVQAGGSLRLSCAASGSISRWLGMGWYRQAPGKEREFVAGITSGANTNYADSVKGRFTISRDNAKNTVYLQMNSLKPEDTAVYYCAAHYVVSVILVYWGQGTQVTVSS
Sim0563 (MRGPRX2 Rank 3)	QVQLQESGGGLVQAGGSLRLSCAASGTIFLPSSMGWYRQAPGKERELVAGITYGAITYYADSVKGRFTISRDNAKNTVYLQMNSLKPEDTAVYYCAVVGLGYGWHFYWGQGTQVTVSS
Sim9877 (MRGPRX2 Rank 5)	QVQLQESGGGLVQAGGSLRLSCAASGYISSFPVMGWYRQAPGKERELVAAIGSGGITYYADSVKGRFTISRDNAKNTVYLQMNSLKPEDTAVYYCAVAGYNIGSYYYWGQGTQVTVSS
Sim9877 I102D	QVQLQESGGGLVQAGGSLRLSCAASGYISSFPVMGWYRQAPGKERELVAAIGSGGITYYADSVKGRFTISRDNAKNTVYLQMNSLKPEDTAVYYCAVAGYN**D**GSYYYWGQGTQVTVSS
Sim9877 Y106A	QVQLQESGGGLVQAGGSLRLSCAASGYISSFPVMGWYRQAPGKERELVAAIGSGGITYYADSVKGRFTISRDNAKNTVYLQMNSLKPEDTAVYYCAVAGYNIGSY**A**YWGQGTQVTVSS
Sim4717 (MRGPRX2 Rank 6)	QVQLQESGGGLVQAGGSLRLSCAASGNIFFYPDMGWYRQAPGKEREFVATIGGGGITYYADSVKGRFTISRDNAKNTVYLQMNSLKPEDTAVYYCAVGGIYVGPHVYWGQGTQVTVSS
Sim4784 (MRGPRX2 Rank 7)	QVQLQESGGGLVQAGGSLRLSCAASGTISPPTYMGWYRQAPGKERELVASIGAGSNTNYADSVKGRFTISRDNAKNTVYLQMNSLKPEDTAVYYCAAIFGRLWYHLYWGQGTQVTVSS
Sim4784 R101E	QVQLQESGGGLVQAGGSLRLSCAASGTISPPTYMGWYRQAPGKERELVASIGAGSNTNYADSVKGRFTISRDNAKNTVYLQMNSLKPEDTAVYYCAAIFG**E**LWYHLYWGQGTQVTVSS

Sim4784 W103D	QVQLQESGGGLVQAGGSLRLSCAASGTISPPTYMGWYRQAPGKERELVASIGAGSNTNYADSVKGRFTISRDNAKNTVYLQMNSLKPEDTAVYYCAAIFGRL**D**YHLYWGQGTQVTVSS
Sim4784 W103G	QVQLQESGGGLVQAGGSLRLSCAASGTISPPTYMGWYRQAPGKERELVASIGAGSNTNYADSVKGRFTISRDNAKNTVYLQMNSLKPEDTAVYYCAAIFGRL**G**YHLYWGQGTQVTVSS
Sim3014 (MRGPRX2 Rank 31)	QVQLQESGGGLVQAGGSLRLSCAASGYISRLGLMGWYRQAPGKEREFVAAISLGSTTYYADSVKGRFTISRDNAKNTVYLQMNSLKPEDTAVYYCAVYNQRRIVDYSNFAYWGQGTQVTVSS
Sim4177 (MRGPRX2 Rank 90)	QVQLQESGGGLVQAGGSLRLSCAASGSISRRGWMGWYRQAPGKEREFVATITFGASTYYADSVKGRFTISRDNAKNTVYLQMNSLKPEDTAVYYCAVFYSQYWLYLYWGQGTQVTVSS
Sim1846 (MRGPRX2 Rank 121)	QVQLQESGGGLVQAGGSLRLSCAASGYIFNATVMGWYRQAPGKERELVATITGGTNTYYADSVKGRFTISRDNAKNTVYLQMNSLKPEDTAVYYCAVVTFVVIPYTYWGQGTQVTVSS
Sim7492 (MRGPRX2 Rank 151)	QVQLQESGGGLVQAGGSLRLSCAASGNIFRIGPMGWYRQAPGKERELVATIASGAITYYADSVKGRFTISRDNAKNTVYLQMNSLKPEDTAVYYCAANHWVASFWYRYLEYWGQGTQVTVSS
Nanobody 60 (Negative Control)	QVQLQESGGGLVQAGGSLRLSCAASGSIFSLNDMGWYRQAPGKLRELVAAITSGGSTKYADSVKGRFTISRDNAKNTVYLQMNSLKAEDTAVYYCNAKVAGTFSIYDYWGQGTQVTVSS

Supplementary Table 2. AlphaFold-Multimer metrics evaluated.

Feature	Description	Included in LCF?
avg_n_contacts	Number of interchain contacts (Cα within ≤10 Å), averaged across five AF-M models	
avg_avg_pAE	PAE scores of interchain contacts, averaged for each model, then averaged across five AF-M models	+
avg_avg_pLDDT	pLDDT scores for interchain contact residues, averaged for each model, then averaged across five AF-M models	+
avg_pDockQ	pDockQ scores, averaged across five AF-M models	
avg_pTM	pTM scores, averaged across five AF-M models	+
avg_ipTM	ipTM scores, averaged across five AF-M models	
avg_rTM	"Ranking TM" scores (rTM = 0.2 * pTM + 0.8 * ipTM), averaged across five AF-M models	
best_model_n_contacts	Number of interchain contacts (Cα within ≤10 Å) for highest ranking AF-M model	
best_model_avg_pAE	Average PAE score of interchain contacts for highest ranking AF-M model	+
best_model_avg_pLDDT	Average pLDDT score for interchain contact residues for highest ranking AF-M model	+
best_model_pDockQ	pDockQ score for highest ranking AF-M model	
best_model_pTM	pTM score for highest ranking AF-M model	+
best_model_ipTM	ipTM score for highest ranking AF-M model	
best_model_rTM	"Ranking TM" score (rTM = 0.2 * pTM + 0.8 * ipTM) for highest ranking AF-M model	
n_unique_contacts	Number of unique interchain contacts (Cα within ≤10 Å), across all five AF-M models	
avg_model_support	Average number of models in which interface contacts are observed	
LCF	Product of six specified features, taken after scaling component features to span the range 0–1	

Supplementary Table 3. Components of Linear Combination Feature (LCF) for virtual screen hits.

Clone	avg_avg_ pAE		avg_avg_ pLDDT		avg_pTM		best_model_ avg_pAE		best_model_ avg_pLDDT		best_model_ pTM		LCF
	Original	Scaled	Original	Scaled	Original	Scaled	Original	Scaled	Original	Scaled	Original	Scaled	
NbSim8619 (rank 1)	5.66	0.82	77.54	0.78	0.79	0.79	4.01	0.87	82.15	0.82	0.82	0.82	0.2962
NbSim7252 (rank2)	5.30	0.83	78.88	0.79	0.80	0.80	5.05	0.84	79.07	0.79	0.78	0.78	0.2713
NbSim0563 (rank3)	5.89	0.81	75.28	0.75	0.78	0.78	4.58	0.86	79.91	0.80	0.81	0.81	0.2642
NbSim9877 (rank5)	5.69	0.82	77.13	0.77	0.80	0.80	5.41	0.83	78.18	0.78	0.78	0.78	0.2562
NbSim4717 (rank6)	7.39	0.77	74.64	0.75	0.76	0.76	4.19	0.87	83.01	0.83	0.81	0.81	0.2534
NbSim4784 (rank7)	6.54	0.79	74.90	0.75	0.76	0.76	4.19	0.87	81.18	0.81	0.79	0.79	0.2523
NbSim3014 (rank31)	6.55	0.79	74.64	0.75	0.78	0.78	5.82	0.82	76.05	0.76	0.80	0.80	0.2308
NbSim4177 (rank90)	7.81	0.75	70.52	0.71	0.75	0.75	5.01	0.84	77.75	0.78	0.80	0.80	0.2083
NbSim1846 (rank121)	7.66	0.76	71.56	0.72	0.76	0.76	5.39	0.83	76.21	0.76	0.77	0.77	0.2006
NbSim7492 (rank151)	8.42	0.73	71.21	0.71	0.73	0.73	5.65	0.82	79.20	0.79	0.78	0.78	0.1940

Supplementary Table 4. Dissociation constants of monomeric nanobodies and point mutants.

	Log Kd ± SEM
Nb Sim8619 WT	-6.3 ± 0.30
Nb Sim8619 Y100G	-2.7 ± 0.24
Nb Sim8619 Y105D	-4.4 ± 1.6
Nb Sim8619 Y105G	-2.6 ± 0.42
Nb Sim9877 WT	-5.0 ± 1.3
Nb Sim9877 I102D	-4.3 ± 2.0
Nb Sim9877 Y106A	-3.9 ± 1.0
Nb Sim4784 WT	-4.2 ± 1.5
Nb Sim4784 R101E	Low binding
Nb Sim4784 W103D	Low binding
Nb Sim4784 W103G	Low binding

SUPPLEMENTARY FIGURES

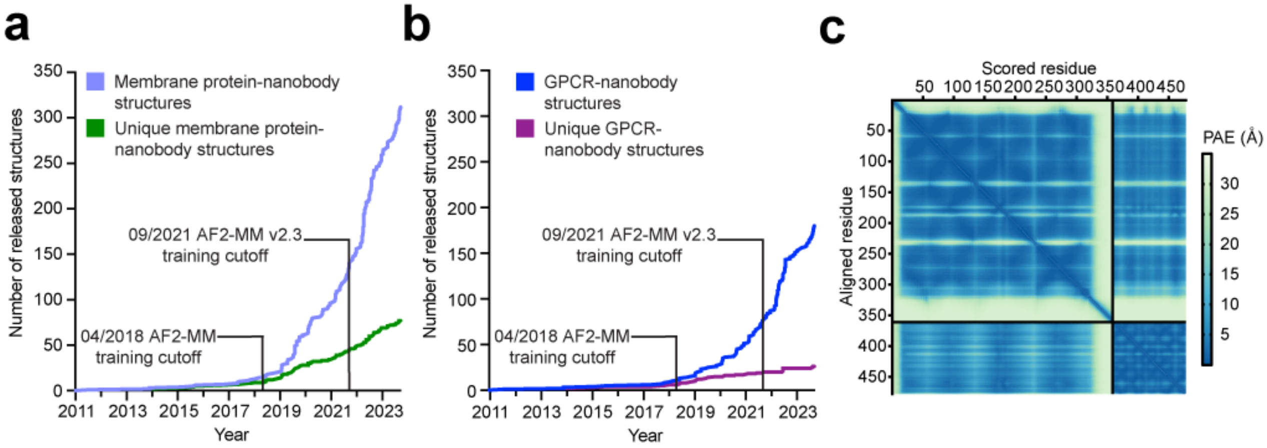

Supplementary Figure 1. a-b). The number of membrane protein-nanobody structures and GPCR-nanobody structures deposited in the PDB over time. c). PAE plot of the AT1R/AT118 complex AF-M prediction. The low PAE values in the AT1R/AT118 binding interface suggest AF-M is highly confident in the binding interaction.

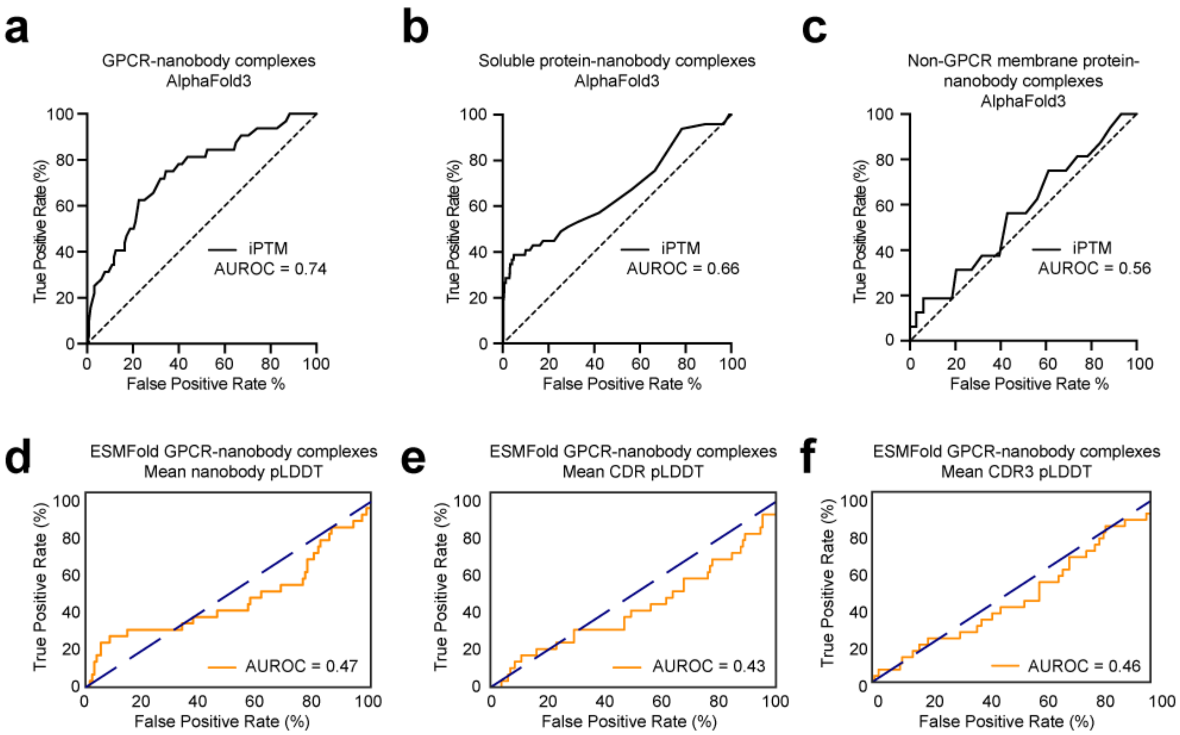

Supplementary Figure 2. (a-c). AlphaFold3 can differentiate between true GPCR-nanobody complexes versus negative controls (a) and more moderately, true soluble protein-nanobody complexes and negative controls (b). AlphaFold3 cannot differentiate between real non-GPCR membrane protein-nanobody complexes and negative controls (c). d-f). ESMFold does not accurately differentiate between true GPCR-nanobody binding interactions and negative control interactions, as assessed by pLDDT values of the entire nanobody (d), the CDR regions (e), and the CDR3 region (f).

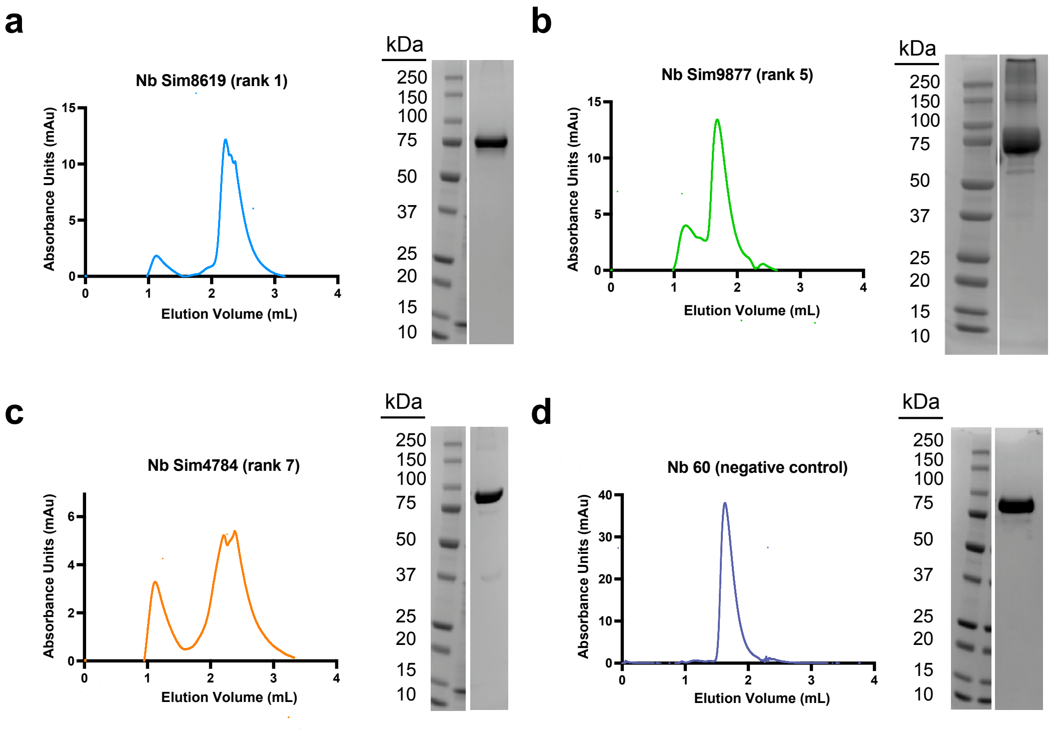

Supplementary Figure 3. a-d). Top-ranked MRGPRX2 simulated nanobodies and the negative control nanobody 60 were expressed and purified from Expi293 cells and then analyzed by analytical size exclusion chromatography, gel electrophoresis, and Coomassie blue staining to assess monodispersity and purity. Nanobodies were run on a Superdex 200 Increase 3.2/300 column.

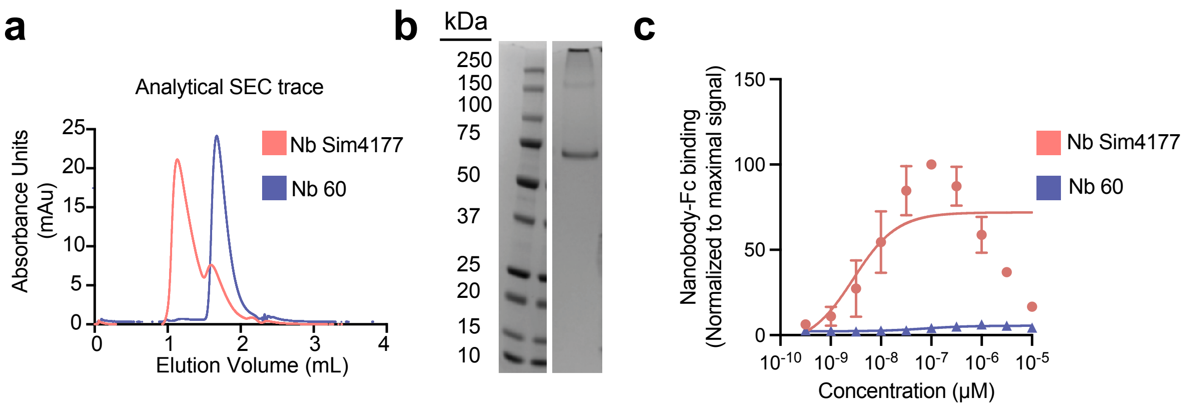

Supplementary Figure 4. a) Size exclusion chromatography trace of nanobody Sim4177 (rank 90) overlayed with the trace of nanobody 60, showing that most of Sim4177 elutes near the void of the column. Nanobodies were run on a Superdex 200 Increase 3.2/300 column. b) Coomassie stained gel of purified nanobody Sim4177, showing the presence of a higher molecular weight species. c) Nanobody Sim4177 binds to ROSA mast cells, but at high concentrations, exhibits lower binding, potentially indicating aggregation. In Panel C, experiments were performed in biological duplicate with error bars representing mean +/- SEM for technical replicates of a representative experiment.

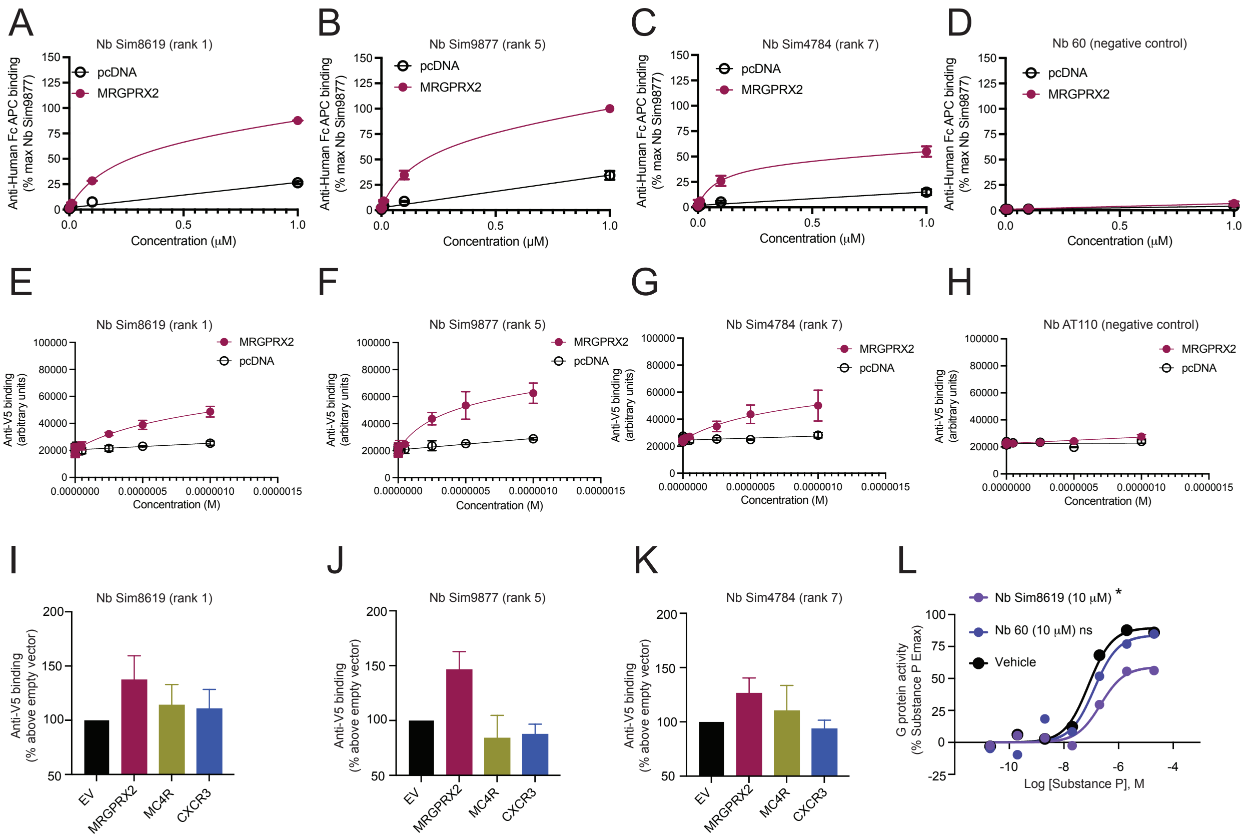

Supplemental Figure 5. HEK293T cells transiently transfected with either human MRGPRX2 or empty vector (pcDNA) were treated for 1 hour at the indicated concentration of the Fc-conjugated a) Sim8619 (rank 1), b) Sim9877 (rank 5), c) Sim4784 (rank 7) or d) the negative control nanobody 60. Similarly, HEK293T cells were transiently transfected with either human MRGPRX2 or empty vector (pcDNA) and treated with monomeric e) Sim8619 (rank 1), f) Sim9877 (rank 5), g) Sim4784 (rank 7), or h) the negative control nanobody AT110 with a C-terminal V5 tag. To assess for promiscuous peptide receptor binding, HEK293T cells transiently transfected with human MRGPRX2, human MC4R, human CXCR3, or empty vector (pcDNA) and treated with at a single high saturating concentration of I) Sim8619 (rank 1), J) Sim9877 (rank 5), K) Sim4784 (rank 7). Experiments were conducted in duplicate or triplicate on separate days, with at least two technical replicates merged per replicate. Error bars indicate mean +/- SEM of three replicates. Dissociation constant and max binding data are available in Table 1 of the main text. l) HEK293T cells overexpressing TRUPATH Gi BRET constructs and WT MRGPRX2 were pretreated with Sim8619 (rank 1), negative control antibody nanobody 60, or vehicle for 45 minutes, and subsequently treated with the indicated concentration of MRGPRX2 peptide agonist substance P. Experiments were performed in technical duplicate or triplicate. Data are normalized to % max signal. *, p<0.05, two-way ANOVA, main effect of pretreatment condition relative to vehicle. Ns, not significant.

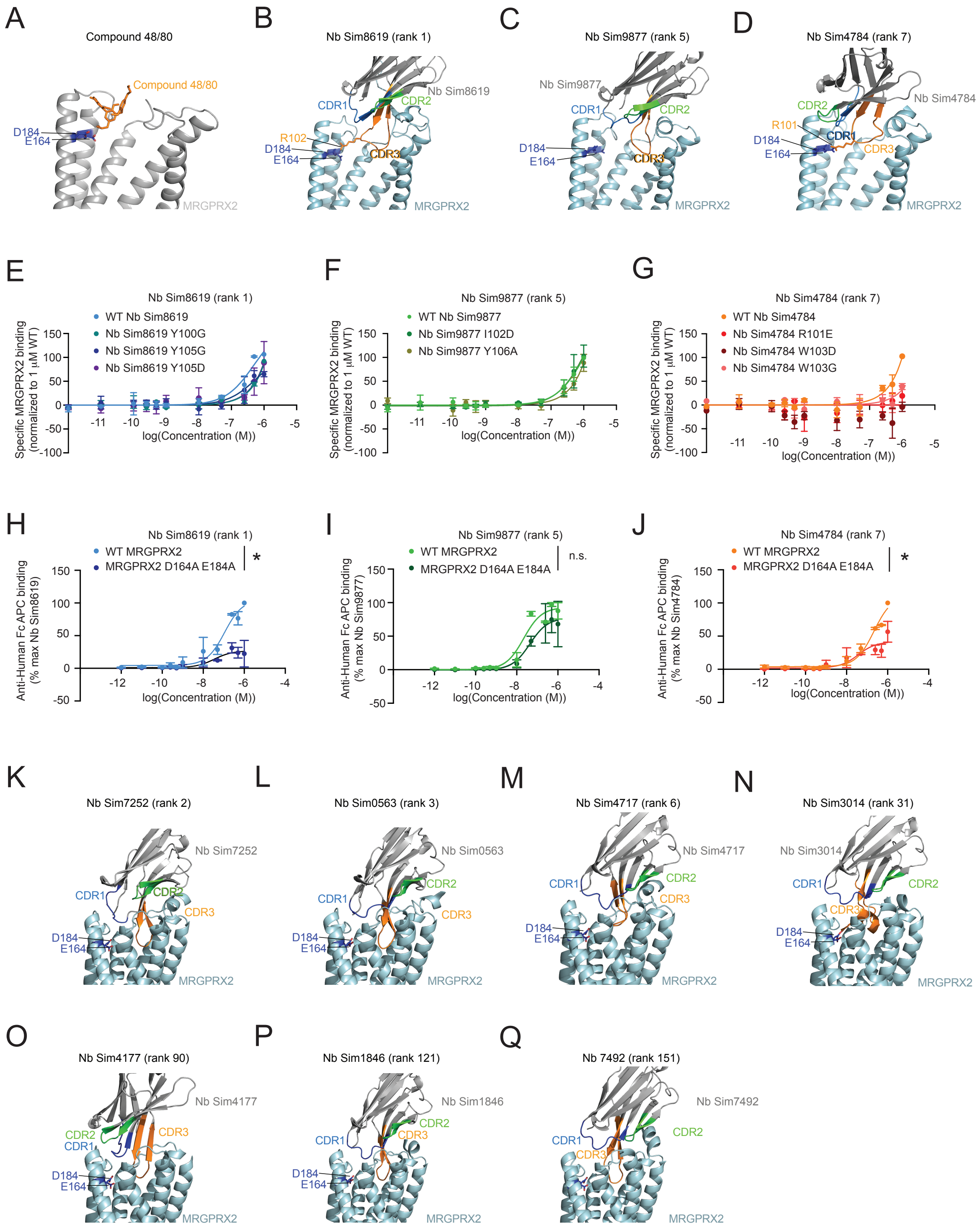

Supplementary Figure 6. a) Structure of Compound 48/80 bound to MRPGRX2 (PDB 7VV6) showing that a positively charged side group of Compound 48/80 interacts with two acidic MRGPRX2 residues. b-d) AlphaFold-Multimer predictions of candidate simulation nanobodies bound to MRGPRX2 that were experimentally confirmed to be true

positive binders. Nanobody Sim8619 (rank 1) and Nanobody 4784 (rank 7) both possess arginine residues in their CDR3 domains that interact with the same two acidic residues in MRGPRX2 that Compound 48/80 interacts with. To assess AlphaFold2 predictions, HEK293T cells were transiently transfected with either human MRGPRX2 or empty vector (pcDNA) were treated for 1 hour at the indicated concentration of monomeric nanobody e) WT Sim8619 (rank 1), f) WT Sim9877 (rank 5), or g) WT Sim4784 (rank 7) and the indicated point mutants. Dissociation constants are available in Supplemental Table 4. Additionally, HEK293 cells were transiently transfected with either human MRGPRX2, MRGPRX2 E164A D184A, or empty vector and were treated for 1 hour at the indicated concentration of the Fc-conjugated h) Sim8619 (rank 1), i) Sim9877 (rank 5), or j). Sim4784 (rank 7). Data were normalized to maximal mean fluorescence intensity signal. pcDNA signal was subtracted from receptor signal and normalized to maximal signal in each respective nanobody's WT MRGPRX2 treatment condition. Experiments were conducted in duplicate on separate days with two technical replicates merged per replicate. *, $p<0.05$, two-way ANOVA, main effect of receptor. ns, not significant. Data shown are mean +/- SEM. k-q) AlphaFold-Multimer predictions of candidate simulation nanobodies bound to MRGPRX2 that were experimentally confirmed not to bind MRGPRX2 in experimental conditions tested.

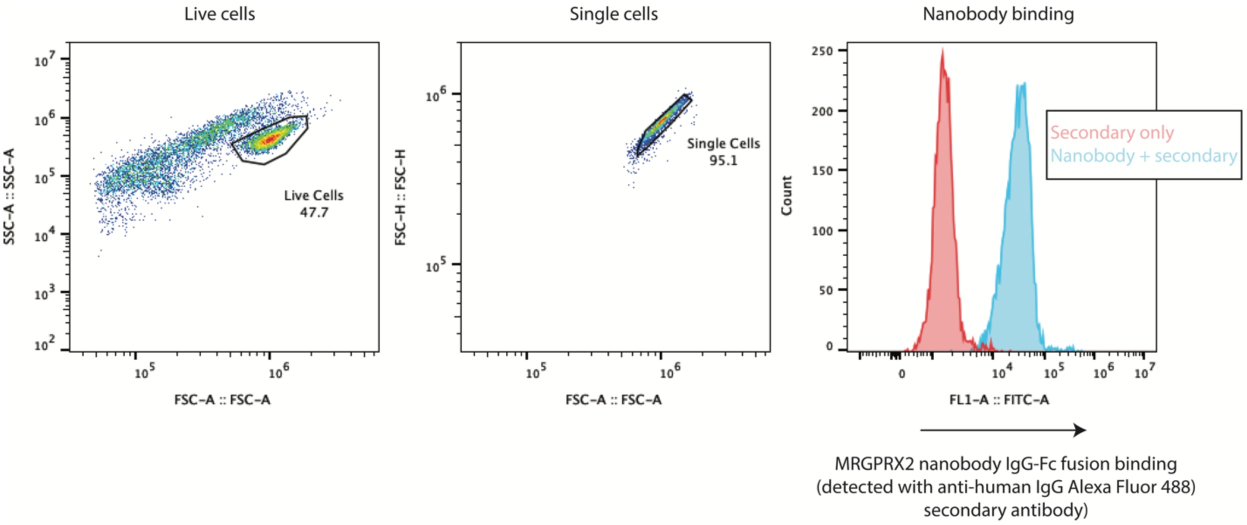

Supplementary Figure 7. Representative gating strategy for mammalian cells. Cells were first gated on live cells and then on singlet cells before analysis of nanobody binding.

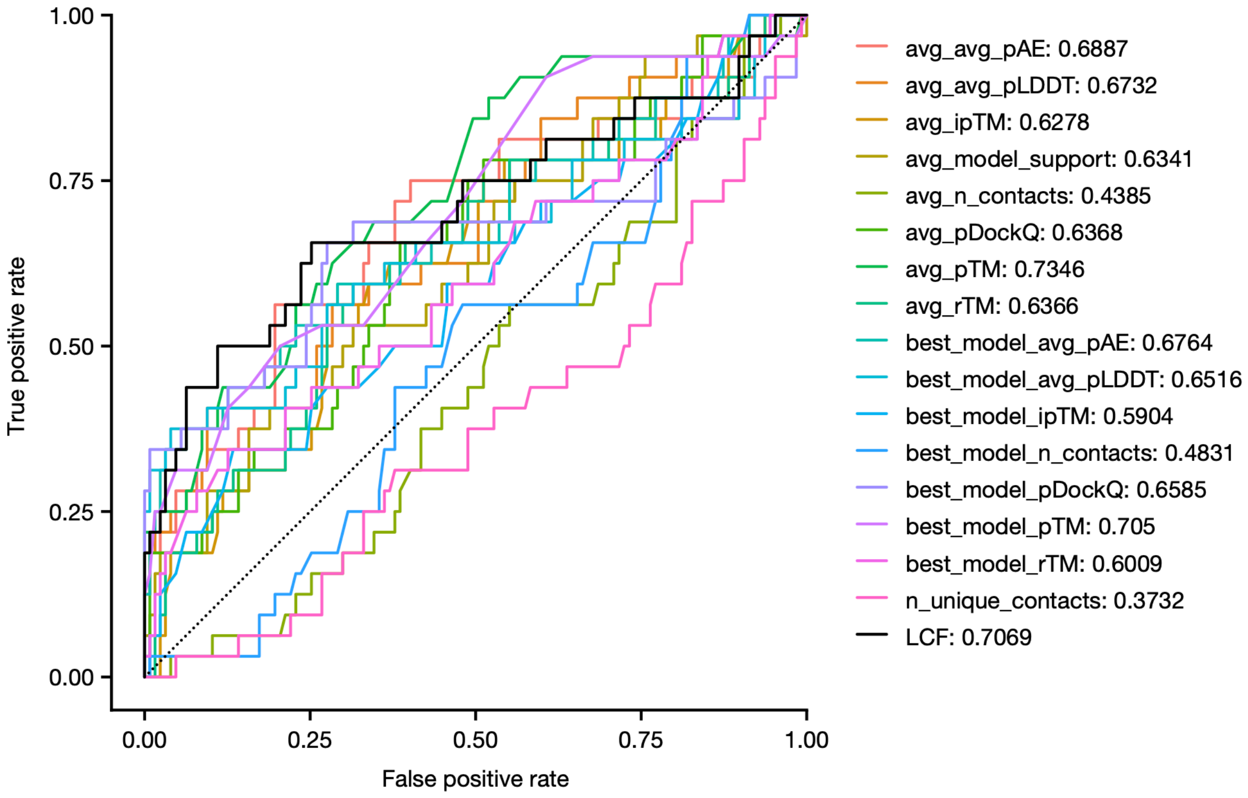

Supplementary Figure 8. AUROC plots for evaluated AF-M features. Receiver operating characteristic (ROC) curves for each evaluated feature on the GPCR-nanobody set are shown.
