# Supplementary material for: In silico discovery of nanobody binders to a G-protein coupled receptor using AlphaFold-Multimer
Source: Nat Commun. 2026 Apr 23;17:5641. doi: 10.1038/s41467-026-72093-5 (PMC13315262; doi:10.1038/s41467-026-72093-5)
Supplement: Supplementary file 2 — Description of Additional Supplementary Files [file 41467_2026_72093_MOESM2_ESM.pdf]

Description of Additional Supplementary Files

File name: Supplementary Data 1
Description: List of nanobody-soluble protein complexes used for benchmarking.

File name: Supplementary Data 2
Description: List of nanobody-GPCR complexes used for benchmarking.

File name: Supplementary Data 3
Description: List of nanobody-non-GPCR complexes used for bechmarking.

File name: Supplementary Data 4
Description: List of sequences of nanobodies in the *in silico* library.

File name: Supplementary Data 5
Description: AlphaFold models of ten experimentally tested nanobodies.
