# Supplementary material for: In silico discovery of nanobody binders to a G-protein coupled receptor using AlphaFold-Multimer
Source: Nat Commun. 2026 Apr 23;17:5641. doi: 10.1038/s41467-026-72093-5 (PMC13315262; doi:10.1038/s41467-026-72093-5)
Supplement: Supplementary file 8 — Reporting Summary [file 41467_2026_72093_MOESM8_ESM.pdf]

nature portfolio

Corresponding author(s): Andrew Kruse and Katherine Susa

Last updated by author(s): Feb 27, 2026

Reporting Summary

Nature Portfolio wishes to improve the reproducibility of the work that we publish. This form provides structure for consistency and transparency in reporting. For further information on Nature Portfolio policies, see our Editorial Policies and the Editorial Policy Checklist.

Statistics

For all statistical analyses, confirm that the following items are present in the figure legend, table legend, main text, or Methods section.

n/a	Confirmed	
☐	☒	The exact sample size (*n*) for each experimental group/condition, given as a discrete number and unit of measurement
☐	☒	A statement on whether measurements were taken from distinct samples or whether the same sample was measured repeatedly
☐	☒	The statistical test(s) used AND whether they are one- or two-sided<br>*Only common tests should be described solely by name; describe more complex techniques in the Methods section.*
☒	☐	A description of all covariates tested
☒	☐	A description of any assumptions or corrections, such as tests of normality and adjustment for multiple comparisons
☐	☒	A full description of the statistical parameters including central tendency (e.g. means) or other basic estimates (e.g. regression coefficient) AND variation (e.g. standard deviation) or associated estimates of uncertainty (e.g. confidence intervals)
☐	☒	For null hypothesis testing, the test statistic (e.g. *F*, *t*, *r*) with confidence intervals, effect sizes, degrees of freedom and *P* value noted<br>*Give P values as exact values whenever suitable.*
☒	☐	For Bayesian analysis, information on the choice of priors and Markov chain Monte Carlo settings
☒	☐	For hierarchical and complex designs, identification of the appropriate level for tests and full reporting of outcomes
☒	☐	Estimates of effect sizes (e.g. Cohen's *d*, Pearson's *r*), indicating how they were calculated

Our web collection on statistics for biologists contains articles on many of the points above.

Software and code

Policy information about availability of computer code

Data collection	Only commercial software was used. Software was supported by the SBGrid consortium. AlphaFold-Multimer predictions were performed using a rented Lamba GPU using ColabFold scripts from https://github.com/YoshitakaMo/localcolabfold. Cytometry data were collected using BD Accuri C6 flow cytometry software (version 1.0.264.21).
Data analysis	Data from cell binding experiments, TruPath assays, mast cell degranulation assays, and other biochemical assays were plotted in GraphPad Prism (version 9.2.0). Code for analyzing AF-M results and ranking nanobody clones can be found on GitHub: https://github.com/kruselab/MRGPRX2-AF-M-screen.

For manuscripts utilizing custom algorithms or software that are central to the research but not yet described in published literature, software must be made available to editors and reviewers. We strongly encourage code deposition in a community repository (e.g. GitHub). See the Nature Portfolio guidelines for submitting code & software for further information.

Data

Policy information about availability of data

All manuscripts must include a data availability statement. This statement should provide the following information, where applicable:

- Accession codes, unique identifiers, or web links for publicly available datasets
- A description of any restrictions on data availability
- For clinical datasets or third party data, please ensure that the statement adheres to our policy

All data generated for the present study are available by request to the corresponding authors.

Research involving human participants, their data, or biological material

Policy information about studies with human participants or human data. See also policy information about sex, gender (identity/presentation), and sexual orientation and race, ethnicity and racism.

Reporting on sex and gender	n/a
Reporting on race, ethnicity, or other socially relevant groupings	n/a
Population characteristics	n/a
Recruitment	n/a
Ethics oversight	n/a

Note that full information on the approval of the study protocol must also be provided in the manuscript.

Field-specific reporting

Please select the one below that is the best fit for your research. If you are not sure, read the appropriate sections before making your selection.

☒ Life sciences ☐ Behavioural & social sciences ☐ Ecological, evolutionary & environmental sciences

For a reference copy of the document with all sections, see nature.com/documents/nr-reporting-summary-flat.pdf

Life sciences study design

All studies must disclose on these points even when the disclosure is negative.

Sample size	All experiments were performed in at least biological duplicates except where noted and sample sizes for each experiment are indicated in the figure legends. We chose sample sizes to ensure reproducibility in accordance to standard operating procedures for each of the experiments.
Data exclusions	No data were excluded from analysis.
Replication	At least two biological duplicate experiments were performed in technical triplicates for all main text experiments. All replicate attempts were successful.
Randomization	n/a
Blinding	n/a

Reporting for specific materials, systems and methods

We require information from authors about some types of materials, experimental systems and methods used in many studies. Here, indicate whether each material, system or method listed is relevant to your study. If you are not sure if a list item applies to your research, read the appropriate section before selecting a response.

Materials & experimental systems

n/a	Involved in the study
☐	☒ Antibodies
☐	☒ Eukaryotic cell lines
☒	☐ Palaeontology and archaeology
☒	☐ Animals and other organisms
☒	☐ Clinical data
☒	☐ Dual use research of concern
☒	☐ Plants

Methods

n/a	Involved in the study
☒	☐ ChIP-seq
☐	☒ Flow cytometry
☒	☐ MRI-based neuroimaging

Antibodies

Antibodies used: MRGPRX2 antibody (Biolegend Cat# 359005, AB_2750139); anti-V5 antibody conjugated to APC (Kruse lab); anti-human IgG Fc-AF488 (Invitrogen Cat# A-11013)

Validation: MRGPRX2 antibody Cat #359005 was experimentally confirmed to bind cell lines expressing high levels of MRGPRX2 but not cell lines with limited MRGPRX2 expression. All secondary antibodies were validated to bind only in the presence of their respective relevant antigen. Additionally, the anti-v5 antibody conjugated to APC and the anti-human IgG Fc-AF488 antibodies were used in our labs successfully in unrelated experiments and the anti-human IgG Fc-AF488 antibody was validated by the manufacturer according to standard protocols.

Eukaryotic cell lines

Policy information about cell lines and Sex and Gender in Research

Cell line source(s): Expi293F (Thermo Fisher Scientific Cat No A14635); HEK293T (ATCC: CRL-3216); the wild type ROSA mast cell line was a gift from the Galli lab (Stanford University).

Authentication: No additional authentication of cell lines was performed.

Mycoplasma contamination: All cell lines tested negative for mycoplasma contamination.

Commonly misidentified lines (See ICLAC register): None were used.

Plants

Seed stocks: n/a

Novel plant genotypes: n/a

Authentication: n/a

Flow Cytometry

Plots

Confirm that:

☒ The axis labels state the marker and fluorochrome used (e.g. CD4-FITC).

☒ The axis scales are clearly visible. Include numbers along axes only for bottom left plot of group (a 'group' is an analysis of identical markers).

☒ All plots are contour plots with outliers or pseudocolor plots.

☒ A numerical value for number of cells or percentage (with statistics) is provided.

Methodology

Sample preparation: All sample preparations for flow cytometry are described in the methods section: "Nanobody-Fc fusion on-cell cytometry

Sample preparation	binding assays" and "Monomeric nanobody on-cell cytometry binding assays."
Instrument	BD Accuri C6 flow cytometer or CytoFLEX S
Software	BD Accuri C6 flow cytometry software (version 1.0.264.21) or CytExpert software (v 1.2)
Cell population abundance	Flow cytometry in our study was not used to determine relative abundances among populations.
Gating strategy	For all flow cytometry experiments, the gating strategies are shown in the extended data figures. For FCS/SSC gating, the central population was gated. To select for single cells, the population forming a uniform population with a 1:1 ratio between FSC-H and FSC-A was gated.

☒ Tick this box to confirm that a figure exemplifying the gating strategy is provided in the Supplementary Information.
