# Supplementary material for: In silico discovery of nanobody binders to a G-protein coupled receptor using AlphaFold-Multimer
Source: Nat Commun. 2026 Apr 23;17:5641. doi: 10.1038/s41467-026-72093-5 (PMC13315262; doi:10.1038/s41467-026-72093-5)
Supplement: Supplementary file 9 — Transparent Peer Review file [file 41467_2026_72093_MOESM9_ESM.pdf]

nature portfolio

Peer Review File

In silico discovery of nanobody binders to a G-protein coupled receptor using AlphaFold-Multimer

Corresponding Author: Dr Katherine Susa

This manuscript has been previously reviewed at another journal. This document only contains information relating to versions considered at Nature Communications.

This file contains all reviewer reports in order by version, followed by all author rebuttals in order by version.

Version 0:

Reviewer comments:

Reviewer #1

(Remarks to the Author)
The authors have addressed all concerns in a constructive way. I agree with the authors that further biochemical/structural experiments with purified MRGPRX2 go beyond the scope of this work, considering the challenges of purifying this receptor. In addition, the authors added some extra cautionary words concerning the expected challenges of the this in silico approach for other GPCRs.
In my view, the paper is ready to be published in this form.

(Remarks on code availability)

Reviewer #2

(Remarks to the Author)
The manuscript by Harvey et al. focuses on the use of AlphaFold-Multimer to discover nanobody binders to G protein-coupled receptors (GPCRs), in particular MRGPRX2. This approach could potentially reduce the extensive experimental efforts required to discover antibodies, which are becoming increasingly important in drug discovery, and such tools would be especially valuable for GPCRs. The authors first explore different metrics to discriminate between interacting and non-interacting nanobodies, followed by a prospective screen of 10,000 nanobodies against MRGPRX2. Experimental evaluation of the top 10 ranked nanobodies led to the discovery of several high-affinity binders. Three nanobodies were also shown to be antagonists with some specificity for MRGPRX2 over two other GPCRs. Mutagenesis of the nanobodies resulted in decreased affinity, providing support for the accuracy of the AlphaFold-Multimer predictions.

The prediction of complexes with antibodies has been a challenging task for AlphaFold and other deep learning methods, so the topic of this work is important. The results of the study are interesting, and I particularly appreciate the prospective screen for novel antibodies. The discovered nanobodies are evaluated using several orthogonal assays, which is also a strength of the work. I have previously reviewed this paper in a different journal, and the authors have addressed the minor points in their response and revised manuscript. However, as a demonstration of AlphaFold's capabilities, the work still has weaknesses.

In my view, one of the following three concerns below needs to be addressed in order for this work to be considered for publication in Nature Communications:

1. Confirmation of the accuracy of the predicted structures: The lack of experimental structures of the GPCR–nanobody complexes is a weakness of the work. Such structures are crucial for confirming that the AlphaFold-Multimer predictions are correct for the right reasons. In addition, access to experimental structures could not only confirm the computational models but also provide insights into the molecular mechanism of antagonism. Similar preprints have solved structures for several GPCR targets to confirm prediction accuracy:

https://www.biorxiv.org/content/10.1101/2025.03.23.644666v2

2. Show that it is plausible that the same approach can be applied to other GPCRs: MRGPRX2 is a promiscuous binder of proteins, so the transferability of the approach to other GPCR targets remains unclear. The paper would be strengthened by demonstrating that the approach can be applied to additional targets. One way to do this would be to perform a prospective screen for another GPCR and identify nanobody binders experimentally. An alternative approach, which is acceptable but less elegant, would be to apply the computational workflow to five additional, diverse GPCRs and assess if AlphaFold-Multimer can identify promising candidates (i.e., with good confidence metrics). The results of these calculations would show the potential of the method and guide the design of future studies using the same technique. A design pipeline applicable to only one receptor would have limited relevance for a broad-readership journal.

3. Make a methodological advance compared to the original AlphaFold implementation: Although the finding that AlphaFold-Multimer can identify nanobody binders of GPCRs is interesting, the work primarily evaluates the performance of the standard AlphaFold-Multimer model and does not present any major methodological advances compared to the original implementation.

Minor comments:

1. I could not find the predicted 3D structures of the predicted nanobodies in the supplementary information. The models of the ten predicted receptor–nanobody complexes should be included with the publication.

(Remarks on code availability)

Reviewer #3

(Remarks to the Author)
This manuscript presents a study on the use of AlphaFold-Multimer (AF-M) for the in silico discovery of nanobodies targeting the MRGPRX2. The identification of functional nanobody is a notable achievement. However, several issues should be addressed to strengthen the claims and improve clarity.

1. The authors demonstrate that AF-M performs well for GPCR-nanobody complexes but poorly for soluble and non-GPCR membrane proteins. The authors are encouraged to explore potential reasons to help readers understand the scope and limitations of the method.

2. While mutational studies support the predicted binding mode, the structural validation remains indirect. The authors are encouraged to include additional experimental evidence to validate the AF-M predictions.

3. The virtual nanobody library was designed based on a previously published yeast display library. The authors should clarify whether this library contains any sequences with known GPCR-binding propensity, and whether the success rate might be influenced by prior structural or sequence biases. A more detailed discussion of library composition and potential biases should be provided.

4. The comparison with AlphaFold3 and ESMFold is valuable but preliminary. A more robust and quantitative comparison should be provided for a fair assessment of model performance.

5. The mechanism of antagonism could be further elucidated. Additional experiments, such as competition binding with known orthosteric ligands or signaling assays with other pathways, would help clarify the mode of inhibition.

(Remarks on code availability)

Version 1:

Reviewer comments:

Reviewer #2

(Remarks to the Author)
I appreciate the authors' efforts to determine structures of the receptor–nanobody complexes. Although these attempts were not successful, the new mutagenesis data provide further support for the complexes predicted by AlphaFold. In addition, the experiments evaluating antagonism using substance P further validates the mechanism of action of the nanobodies. Taken together, I consider the new data sufficient to justify publication in Nature Communications.

If not already included, the authors should provide the PDBs of the 10 predicted receptor–nanobody complexes as supplementary files. The manuscript cannot be published without this data, as it provides others the opportunity to assess the quality of the models.

(Remarks on code availability)

Reviewer #3

(Remarks to the Author)
The manuscript has been strengthened by the additional clarifications, data, and discussion provided. I find that the revisions adequately address my concerns.

(Remarks on code availability)
Sufficient

Open Access This Peer Review File is licensed under a Creative Commons Attribution 4.0 International License, which permits use, sharing, adaptation, distribution and reproduction in any medium or format, as long as you give appropriate credit to the original author(s) and the source, provide a link to the Creative Commons license, and indicate if changes were made.
In cases where reviewers are anonymous, credit should be given to 'Anonymous Referee' and the source.
The images or other third party material in this Peer Review File are included in the article's Creative Commons license, unless indicated otherwise in a credit line to the material. If material is not included in the article's Creative Commons license and your intended use is not permitted by statutory regulation or exceeds the permitted use, you will need to obtain permission directly from the copyright holder.
To view a copy of this license, visit https://creativecommons.org/licenses/by/4.0/

REVIEWER COMMENTS

Reviewer #1 (Remarks to the Author):

The authors have addressed all concerns in a constructive way. I agree with the authors that further biochemical/structural experiments with purified MRGPRX2 go beyond the scope of this work, considering the challenges of purifying this receptor. In addition, the authors added some extra cautionary words concerning the expected challenges of the this in silico approach for other GPCRs.
In my view, the paper is ready to be published in this form.

We thank the reviewer for his/her helpful suggestions, the time spent evaluating our manuscript, and for recognizing the efforts made to improve it during revision.

Reviewer #2 (Remarks to the Author):

The manuscript by Harvey et al. focuses on the use of AlphaFold-Multimer to discover nanobody binders to G protein-coupled receptors (GPCRs), in particular MRGPRX2. This approach could potentially reduce the extensive experimental efforts required to discover antibodies, which are becoming increasingly important in drug discovery, and such tools would be especially valuable for GPCRs. The authors first explore different metrics to discriminate between interacting and non-interacting nanobodies, followed by a prospective screen of 10,000 nanobodies against MRGPRX2. Experimental evaluation of the top 10 ranked nanobodies led to the discovery of several high-affinity binders. Three nanobodies were also shown to be antagonists with some specificity for MRGPRX2 over two other GPCRs. Mutagenesis of the nanobodies resulted in decreased affinity, providing support for the accuracy of the AlphaFold-Multimer predictions.

The prediction of complexes with antibodies has been a challenging task for AlphaFold and other deep learning methods, so the topic of this work is important. The results of the study are interesting, and I particularly appreciate the prospective screen for novel antibodies. The discovered nanobodies are evaluated using several orthogonal assays, which is also a strength of the work. I have previously reviewed this paper in a different journal, and the authors have addressed the minor points in their response and revised manuscript. However, as a demonstration of AlphaFold's capabilities, the work still has weaknesses.

In my view, one of the following three concerns below needs to be addressed in order for this work to be considered for publication in Nature Communications:

1. Confirmation of the accuracy of the predicted structures: The lack of experimental structures of the GPCR–nanobody complexes is a weakness of the work. Such structures are crucial for confirming that the AlphaFold-Multimer predictions are correct for the right reasons. In addition, access to experimental structures could not only confirm the computational models but also provide insights into the molecular mechanism of antagonism. Similar preprints have solved structures for several GPCR targets to confirm prediction accuracy:
https://www.biorxiv.org/content/10.1101/2025.03.23.644666v2

We appreciate the reviewer's emphasis on the value of experimental GPCR–nanobody structures. As noted, our primary contribution here is to demonstrate that AlphaFold-Multimer enables the rapid identification of functional nanobody binders, thereby

bypassing several resource-intensive experimental steps historically required for early-stage nanobody/antibody discovery. Importantly, the central conclusions of the manuscript do not depend on the availability of an experimental structure. This is a critical point, as many of the most therapeutically relevant receptors lack relevant structural data, both by themselves and in complex with antibody binders.

We fully agree that a high-resolution structure of MRGPRX2 in complex with one of our nanobodies would be of great interest. We did pursue purification of MRGPRX2 for this purpose, but this receptor presents biochemical challenges that precluded obtaining material suitable for structural analysis within a reasonable timeframe. Therefore, determining such a structure is outside the scope of the present study.

We have included size-exclusion traces of our purification attempts below. These traces show that the vast majority of MRGPRX2 eluted in the void peak of the column, indicating aggregated receptor that is not amenable to structural biology.

Purification attempt 1:

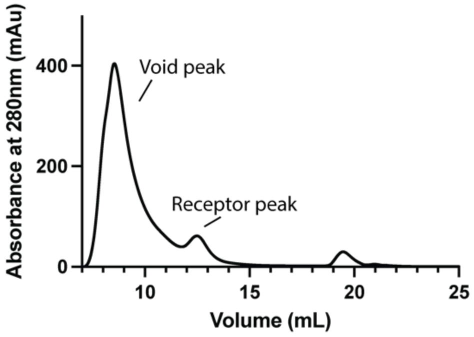

Purification attempt 2:

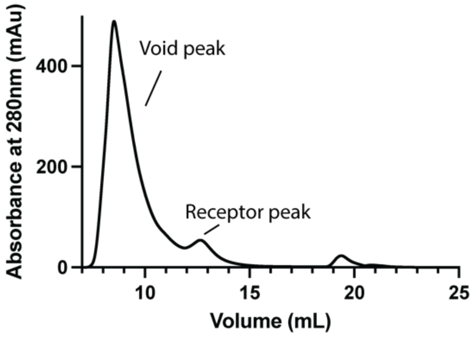

Considering our difficulty producing MRGPRX2 for structural work, we have instead included extensive mutagenesis to validate the binding pose of our nanobodies. In previous versions of our manuscript, we had included mutagenesis data in the Main Text Figure 5E and Sup. Figure 6 Panels E-G. For these experiments, we expressed and purified our nanobodies with point mutations within their CDRs at positions predicted by the AlphaFold-Multimer model to interact with MRGPRX2. In support of the predicted binding pose, these mutant nanobodies bound to MRGPRX2 with a decrease in apparent affinity, which is summarized in Supplemental Table 4.

To further support the predicted binding poses, we have now added additional mutagenesis data during this round of revision. In the AlphaFold-Multimer models of Nb Sim8619 (rank 1) and Sim4787 (rank 7), an arginine residue within CDR3 and is predicted to form a salt bridge with D184 and E164 of MRGPRX2, while the CDR3 of Sim 9877 (rank 5) is not predicted to make significant contacts with D184/E164 (shown in Sup. Figure 6 B-D). In Sup. Figure 6 Panels H-J, we now show data testing the effects of mutating D184 and E164 to alanine on Nb Sim8619 (rank 1), Sim9877 (rank 5), and Sim4784 (rank 7) binding. As expected, Nb Sim8619 (rank 1) and Nb Sim4784 (rank 7) bound MRGPRX2 E164A/D184A with a significantly lower Bmax compared to wild-type MRGPRX2, indicating that the mutations result in a lower percentage of MRPGRX2 that is amenable to nanobody binding. On the other hand, Nb Sim9877 (rank 5) showed no significant difference in binding wild-type MRGPRX2 vs. MRGPRX2 with the E164A/D184A mutations, further supporting the predicted binding poses. We have updated lines 405-414 of the main text to explain these results.

We have also expanded our Discussion to explain the challenges we encountered in purifying MRGPRX2 for structural work and to note that, although we performed extensive mutagenesis, the precise binding pose and mode of inhibition of our nanobodies cannot be determined without an experimental structure (lines 492-496).

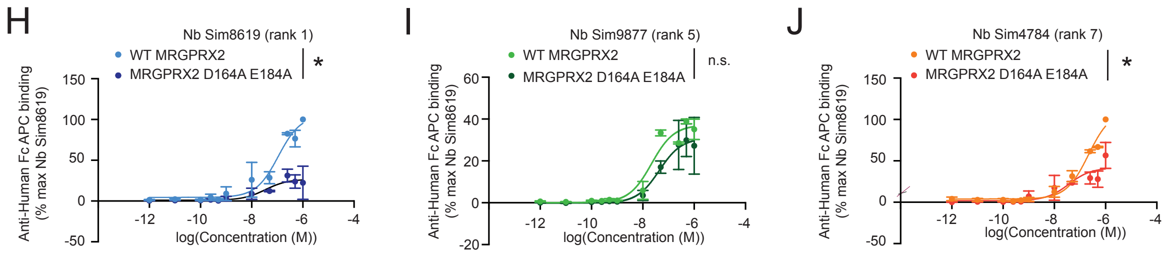

Supplementary Figure 6 H-J:
Additionally, HEK293 cells were transiently transfected with either human MRGPRX2, MRGPRX2 E164A D184A, or empty vector and were treated for 1 hour at the indicated concentration of the Fc-conjugated h) Sim8619 (rank 1), i) Sim9877 (rank 5), or j). Sim4784 (rank 7). Data were normalized to maximal mean fluorescence intensity signal. pcDNA signal was subtracted from receptor signal and normalized to maximal signal in each respective nanobody's WT MRGPRX2 treatment condition. Experiments were conducted in duplicate on separate days with two technical replicates merged per replicate. *, $p<0.05$, two-way ANOVA, main effect of receptor. ns, not significant. Data shown are mean +/- SEM.

2. Show that it is plausible that the same approach can be applied to other GPCRs: MRGPRX2 is a promiscuous binder of proteins, so the transferability of the approach to other GPCR targets remains unclear. The paper would be strengthened by demonstrating that the approach can be applied to additional targets. One way to do this would be to perform a prospective screen for another GPCR and identify nanobody binders experimentally. An alternative approach, which is acceptable but less elegant, would be to apply the computational workflow to five additional, diverse GPCRs and assess if AlphaFold-Multimer can identify promising candidates (i.e., with good confidence metrics). The results of these calculations would show the potential of the method and guide the design of future studies using the same technique. A design pipeline applicable to only one receptor would have limited relevance for a broad-readership journal.

We appreciate that a key question that comes from our work is to understand if this approach can be applied to other GPCRs. We are excited to investigate this in future studies. MRGPRX2 was chosen both due to its important role in mast cell biology, and also due to its relatively shallow binding pocket, making it an ideal test case for this

approach. As discussed in our manuscript, future efforts targeting other GPCRs will require more computationally intensive resources and library sizes. In response to this comment, we have also updated our discussion (lines 509-517). Here, we have now framed this paragraph to clarify that applying our AF-M screening approach to other GPCRs or receptor types is an important and exciting future question that remains to be solved. We also reference recent advances in computational models that have enabled *in silico* discovery of nanobody binders to other GPCRs and receptors, highlighting the rapid progress the field is making on this difficult challenge.

3. Make a methodological advance compared to the original AlphaFold implementation: Although the finding that AlphaFold-Multimer can identify nanobody binders of GPCRs is interesting, the work primarily evaluates the performance of the standard AlphaFold-Multimer model and does not present any major methodological advances compared to the original implementation.

We appreciate this point but wish to clarify that our work is not an advance in protein structure prediction, but rather the finding that these methods can be judiciously used for prospective screening. We respectfully believe that the reviewer has misunderstood the central message of our manuscript.

Minor comments:

1. I could not find the predicted 3D structures of the predicted nanobodies in the supplementary information. The models of the ten predicted receptor–nanobody complexes should be included with the publication.

We thank the reviewer for this suggestion, and we have modified Supplementary Figure 6 B-D and K-Q to show all ten predicted receptor-nanobody complexes.

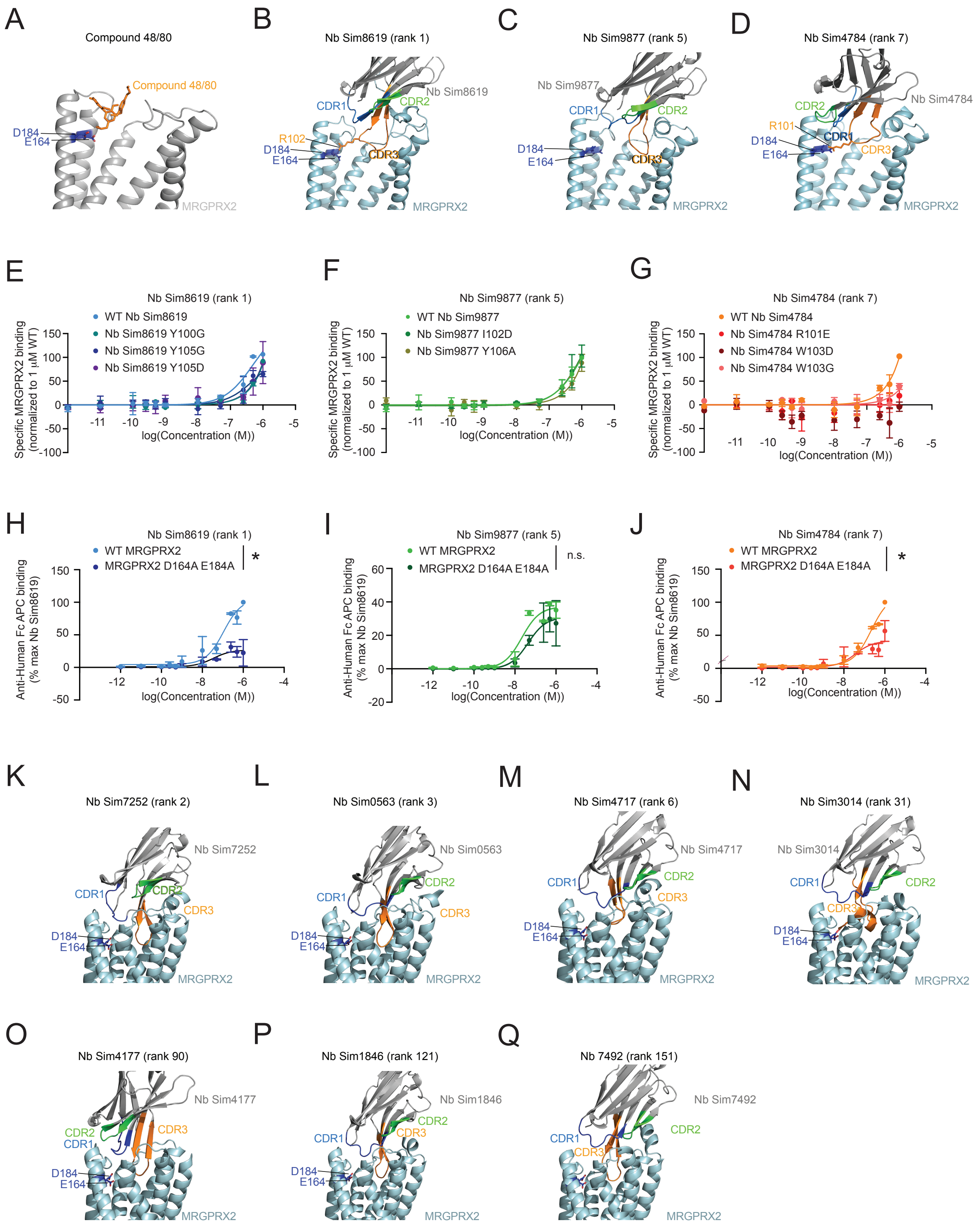

A
Compound 48/80
Compound 48/80
D184
E164
MRGPRX2
B
Nb Sim8619 (rank 1)
Nb Sim8619
CDR1
CDR2
R102
D184
E164
CDR3
MRGPRX2
C
Nb Sim9877 (rank 5)
Nb Sim9877
CDR1
CDR2
D184
E164
CDR3
MRGPRX2
D
Nb Sim4784 (rank 7)
Nb Sim4784
CDR2
R101
CDR1
D184
E164
CDR3
MRGPRX2
E
Nb Sim8619 (rank 1)
WT Nb Sim8619
Nb Sim8619 Y100G
Nb Sim8619 Y105G
Nb Sim8619 Y105D
Specific MRGPRX2 binding (normalized to 1 µM WT)
log(Concentration (M))
F
Nb Sim9877 (rank 5)
WT Nb Sim9877
Nb Sim9877 I102D
Nb Sim9877 Y106A
Specific MRGPRX2 binding (normalized to 1 µM WT)
log(Concentration (M))
G
Nb Sim4784 (rank 7)
WT Nb Sim4784
Nb Sim4784 R101E
Nb Sim4784 W103D
Nb Sim4784 W103G
Specific MRGPRX2 binding (normalized to 1 µM WT)
log(Concentration (M))
H
Nb Sim8619 (rank 1)
WT MRGPRX2
MRGPRX2 D164A E184A
*
Anti-Human Fc APC binding (% max Nb Sim8619)
log(Concentration (M))
I
Nb Sim9877 (rank 5)
WT MRGPRX2
MRGPRX2 D164A E184A
n.s.
Anti-Human Fc APC binding (% max Nb Sim8619)
log(Concentration (M))
J
Nb Sim4784 (rank 7)
WT MRGPRX2
MRGPRX2 D164A E184A
*
Anti-Human Fc APC binding (% max Nb Sim8619)
log(Concentration (M))
K
Nb Sim7252 (rank 2)
Nb Sim7252
CDR1
CDR2
CDR3
D184
E164
MRGPRX2
L
Nb Sim0563 (rank 3)
Nb Sim0563
CDR2
CDR1
CDR3
D184
E164
MRGPRX2
M
Nb Sim4717 (rank 6)
Nb Sim4717
CDR1
CDR2
CDR3
D184
E164
MRGPRX2
N
Nb Sim3014 (rank 31)
Nb Sim3014
CDR1
CDR2
CDR3
D184
E164
MRGPRX2
O
Nb Sim4177 (rank 90)
Nb Sim4177
CDR2
CDR1
CDR3
D184
E164
MRGPRX2
P
Nb Sim1846 (rank 121)
Nb Sim1846
CDR2
CDR1
CDR3
D184
E164
MRGPRX2
Q
Nb 7492 (rank 151)
Nb Sim7492
CDR1
CDR2
CDR3
MRGPRX2

Reviewer #3 (Remarks to the Author):

This manuscript presents a study on the use of AlphaFold-Multimer (AF-M) for the in silico discovery of nanobodies targeting the MRGPRX2. The identification of functional nanobody is a notable achievement. However, several issues should be addressed to strengthen the claims and improve clarity.

1. The authors demonstrate that AF-M performs well for GPCR-nanobody complexes but poorly for soluble and non-GPCR membrane proteins. The authors are encouraged to explore potential reasons to help readers understand the scope and limitations of the method.

We appreciate this point. AF-M likely performs well for GPCR-nanobody complexes due to the relatively large number of nanobody-GPCR structures found in the PDB and the stereotyped binding poses nanobodies use to engage GPCRs. We have updated lines 193-203 to clarify this.

2. While mutational studies support the predicted binding mode, the structural validation remains indirect. The authors are encouraged to include additional experimental evidence to validate the AF-M predictions.

We thank the reviewer for this suggestion. We note that we have included extensive mutagenesis to validate the predicted binding pose of our nanobodies in both Main Text Figure 5 and Supplementary Figure 6. As discussed above (Reviewer 1, comment 1), we have added additional mutagenesis data during this round of revision (Figure 6H-J), and the central conclusions of the manuscript do not depend on the availability of an experimental structure. Determination of a structure of a nanobody bound to MRGPRX2 is out of scope for this manuscript, and we have now also included in our Discussion an explanation of the challenges we encountered in purifying MRGPRX2 for structural work and note that, although we performed extensive mutagenesis, the precise binding pose and mode of inhibition of our nanobodies cannot be determined without an experimental structure (lines 492-496).

3. The virtual nanobody library was designed based on a previously published yeast display library. The authors should clarify whether this library contains any sequences with known GPCR-binding propensity, and whether the success rate might be influenced by prior structural or sequence biases. A more detailed discussion of library composition and potential biases should be provided.

We have updated lines 270-275 to clarify that our library did not contain sequences with known GPCR binding propensity and have included a more detailed discussion of the library composition. We have also included a new Extended Data Table 4, with the sequences of all nanobodies in the in silico library.

4. The comparison with AlphaFold3 and ESMFold is valuable but preliminary. A more robust and quantitative comparison should be provided for a fair assessment of model performance.

We previously included ROC plots in Supplementary Figure 2 illustrating that the primary output metric from ESMFold (pLDDT) does not predict nanobody binding to GPCRs, non-GPCR membrane proteins, or soluble proteins (AUC values range from 0.43–0.47).

AlphaFold3, in contrast, shows performance similar to AlphaFold-Multimer; that is, it is moderately predictive of binding to GPCRs (AUC = 0.74) but has poor accuracy for non-GPCR membrane proteins (AUC = 0.66) and soluble proteins (AUC = 0.56).

The field is rapidly advancing, and high throughput screening with AlphaFold3 has now been made possible by the release of AlphaFold3 code. Unfortunately, this was not the case at the time of submission of this manuscript, and therefore, further investigation of the suitability of AF3 for prospective screening was not possible.

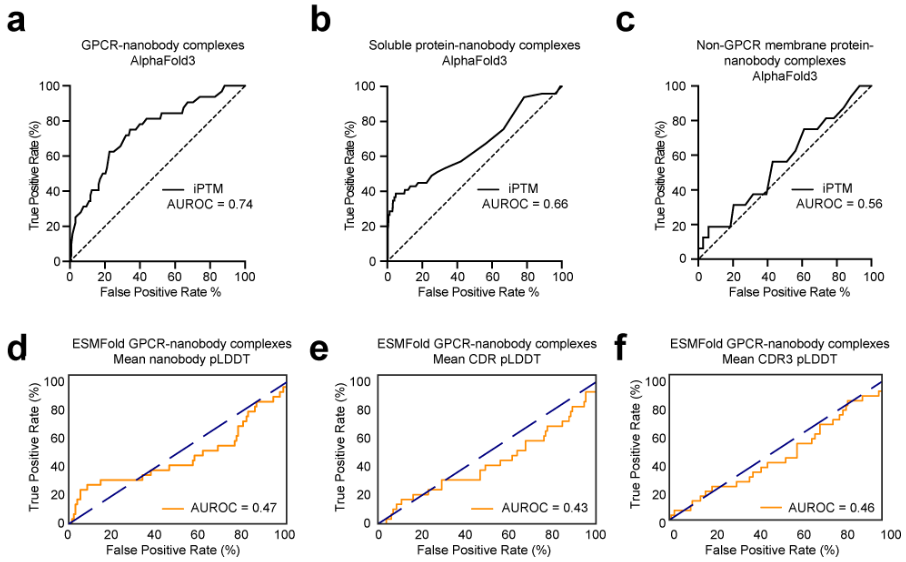

Supplementary Figure 2. (a-c). AlphaFold3 can differentiate between true GPCR-nanobody complexes versus negative controls (a) and more moderately, true soluble protein-nanobody complexes and negative controls (b). AlphaFold3 cannot differentiate between real non-GPCR membrane protein-nanobody complexes and negative controls (c). d-f). ESMFold does not accurately differentiate between true GPCR-nanobody binding interactions and negative control interactions, as assessed by pLDDT values of the entire nanobody (d), the CDR regions (e), and the CDR3 region (f).

5. The mechanism of antagonism could be further elucidated. Additional experiments, such as competition binding with known orthosteric ligands or signaling assays with other pathways, would help clarify the mode of inhibition

We thank the reviewer for this suggestion. We have expanded the mechanistic characterization of antagonism. Previously, in main text Figure 5H, we demonstrated that pretreatment with Sim8619 (Nb Rank 1) produces a rightward shift in compound 48/80–induced G-protein activation, consistent with competitive inhibition.

We have now included analogous experiments using the orthosteric peptide agonist substance P, a peptide ligand that binds in the same shallow binding pocket of MRGPRX2 as Compound 48/80 (PDB 7VDM). Pre-treatment with Sim8619 (Rank 1) similarly shifts the substance P dose-response curve, further supporting that Sim8619 is a competitive binder with known orthosteric ligands. We have added this data to Supplementary Figure 5L and have updated lines 432-438 of the text to explain this result.

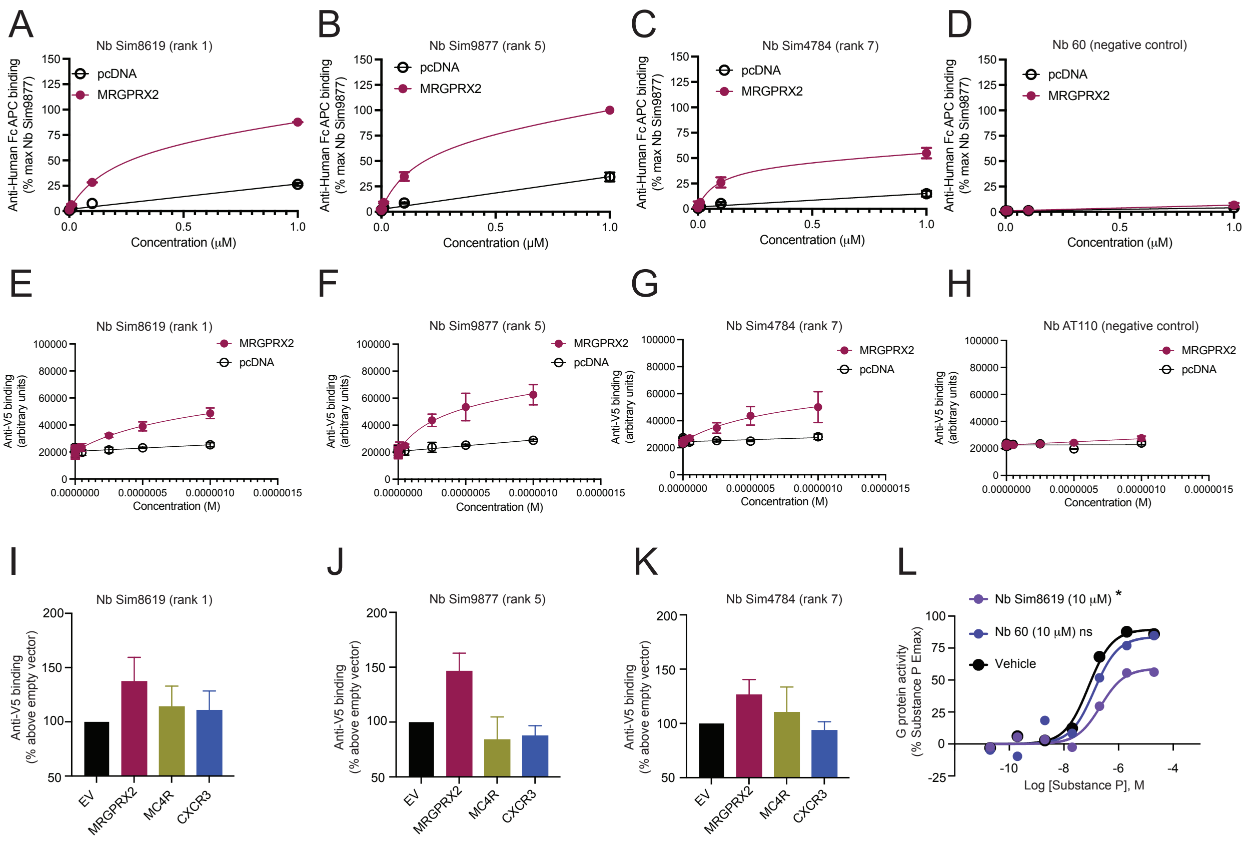

Supplemental Figure 5. HEK293T cells transiently transfected with either human MRGPRX2 or empty vector (pcDNA) were treated for 1 hour at the indicated concentration of the Fc-conjugated a) Sim8619 (rank 1), b) Sim9877 (rank 5), c) Sim4784 (rank 7) or d) the negative control nanobody 60. Similarly in HEK293T cells were transiently transfected with either human MRGPRX2 or empty vector (pcDNA) and treated with monomeric e) Sim8619 (rank 1), f) Sim9877 (rank 5), g) Sim4784 (rank 7) or h) the negative control nanobody AT110 with a C-terminal V5 tag. To assess for promiscuous peptide receptor binding, HEK293T cells transiently transfected with human MRGPRX2, human MC4R, human CXCR3, or empty vector (pcDNA) and treated with at a single high saturating concentration of I) Sim8619 (rank 1), J) Sim9877 (rank 5), K) Sim4784 (rank 7). Experiments were conducted in duplicate or triplicate on separate days, with at least two technical replicates merged per replicate. Dissociation constant and max binding data are available in Table 1 of the main text. L). HEK293T cells overexpressing TRUPATH Gi BRET constructs and WT MRGPRX2 were pretreated with Sim8619 (rank 1), negative control antibody nanobody 60, or vehicle for 45 minutes, and subsequently treated with the indicated concentration of MRGPRX2 peptide agonist substance P. Experiments were performed in technical duplicate or triplicate. Data are normalized to % max signal. *, $p<0.05$, two-way ANOVA, main effect of pretreatment condition relative to vehicle. Ns, not significant. Error bars indicate mean +/- SEM of three replicates.

REVIEWERS' COMMENTS

Reviewer #2 (Remarks to the Author):

I appreciate the authors' efforts to determine structures of the receptor–nanobody complexes. Although these attempts were not successful, the new mutagenesis data provide further support for the complexes predicted by AlphaFold. In addition, the experiments evaluating antagonism using substance P further validates the mechanism of action of the nanobodies. Taken together, I consider the new data sufficient to justify publication in Nature Communications.

We thank the reviewer for their time evaluating our manuscript and for their constructive comments during the review process.

If not already included, the authors should provide the PDBs of the 10 predicted receptor–nanobody complexes as supplementary files. The manuscript cannot be published without this data, as it provides others the opportunity to assess the quality of the models.

Thank you for this suggestion. We have added a Supplemental file with the PDB files of all ten predicted nanobody complexes.

Reviewer #3 (Remarks to the Author):

The manuscript has been strengthened by the additional clarifications, data, and discussion provided. I find that the revisions adequately address my concerns.

We thank the reviewer for their helpful feedback and for the time they spent reviewing our manuscript.

Reviewer #3 (Remarks on code availability):

Sufficient
